# Global Assessment of Environmental and Plant‐Trait Influences on Root: Shoot Biomass Ratios

**DOI:** 10.1111/gcb.70543

**Published:** 2025-10-07

**Authors:** Ruijie Ding, Rodolfo L. B. Nóbrega, Iain Colin Prentice

**Affiliations:** ^1^ Department of Life Sciences, Imperial College London Georgina Mace Centre for the Living Planet Ascot UK; ^2^ School of Geographical Sciences University of Bristol Bristol UK; ^3^ Cabot Institute for the Environment University of Bristol Bristol UK; ^4^ Department of Earth System Science Tsinghua University Beijing China

**Keywords:** carbon allocation, eco‐evolutionary optimality, edaphic factors, gross primary production, plant strategy, plant traits, root: shoot ratio, root‐zone water capacity

## Abstract

The distribution of assimilated carbon (C) among roots, stems, and leaves is a central process in terrestrial ecosystem dynamics. Yet the biomass allocation schemes used in current global vegetation and land surface models pre‐date the existence of large plant‐trait data sets and remain largely untested. Here we formulate hypotheses on the controls of root: shoot biomass ratios (R:S), based on eco‐evolutionary optimality principles, and assess them quantitatively by analysing data on nearly 30,000 observations of R:S. We analysed global R:S patterns using multiple linear regression models for woody and herbaceous species separately, considering as candidate predictors growing‐season mean temperature (*T*_g_), gross primary production (GPP), a measure of root‐zone water capacity (RZ_WC_), soil pH, sand content, aridity index (AI), and plant traits: vegetation height (H), leaf thickness (LT), leaf dry matter content (LDMC), specific leaf area (SLA), specific root length (SRL), and rooting depth (RRD). R:S was systematically greater in herbaceous plants. R:S decreased with *T*_g_, GPP, and height but increased with sand content, RRD, and LDMC in both woody and herbaceous plants. However, AI and leaf thickness had opposing effects on R:S. RZ_WC_ and SLA were important in woody plants, while pH and SRL played a larger role in herbaceous plants. The models explained 13% (woody) and 31% (herbaceous) of R:S variation. The lower explanatory power for woody plants is likely influenced by unmeasured variations in (for example) forest age and canopy position. These empirical findings provide a step towards a quantitative theory of plant C allocation responses to resource availability and an improved C allocation scheme for ecosystem models.

## Graphical abstract

We assembled a large global dataset by combining several previous compilations to test eco‐evolutionary optimality (EEO) hypotheses about how plants allocate carbon above‐ and below‐ground (root: shoot biomass ratios). Our analysis shows how carbon allocation in woody and herbaceous plants responds to environmental conditions and plant traits. These empirical findings provide a step toward a quantitative theory of plant carbon allocation and improved schemes for representing carbon dynamics in ecosystem models.

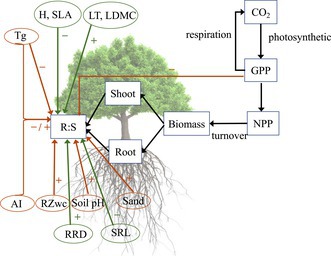

## 1 Introduction

Carbon (C) allocation in plants involves the partitioning of photosynthate into different functional pools: leaves, woody tissues, roots, and reproductive organs. It has long been accepted that C allocation represents a form of economic optimization, avoiding wasteful imbalances between the supply of different resources required for growth (Bloom et al. 1985). In particular, the concept of “functional balance” has been used to explain the partitioning of C between above‐ground and below‐ground organs (Brouwer 1963; Lambers 1983). Functional balance means that plants shift C allocation towards shoots when above‐ground resources (light and CO_2_) are more limiting and towards roots when below‐ground resources (nutrients and water) are more limiting. Such shifts are beneficial because they allow plants to capture more of the resources that most strongly limit their growth Poorter and Nagel 2000).

Optimal partitioning theory is an extension of the functional balance concept. It predicts that plants should allocate C in such a way that all required resources limit growth to the same extent (Bloom et al. 1985; Merganičová et al. 2019; Poorter et al. 2012; Potkay et al. 2021). This arrangement is optimal in the sense that it maximizes the conversion of photosynthate into plant growth. Any other arrangement would imply that some C is being expended to acquire resources that cannot be used. Its predictions have been upheld, at least qualitatively, in experiments. High‐nutrient‐grown plants developed lower root:shoot biomass ratios (R:S) than low‐nutrient‐grown plants (e.g., Gedroc et al. 1996). Meta‐analysis of experimental data confirmed that root biomass increases, at the expense of stems and leaves, when plants are subjected to severe drought or low nutrient supplies, while leaf mass fraction increases with nutrient supplies and decreases with light (Poorter et al. 2012). In arid, cold and/or infertile environments, more biomass is typically partitioned to roots and less to stems and leaves (Gill and Finzi 2016; Hui and Jackson 2006; Reich et al. 2014).

Biomass allocation in plants is closely linked to functional traits that govern resource acquisition, growth strategies, and environmental adaptation (Freschet et al. 2021). Root morphological traits, such as specific root length (SRL), reflect below‐ground allocation strategies for soil exploration and resource uptake efficiency (Freschet and Roumet 2017). Leaf traits, including specific leaf area (SLA) and leaf dry matter content (LDMC), influence C gain and growth rates, with higher SLA and lower leaf thickness supporting faster growth (Pietsch et al. 2014; Poorter and Nagel 2000). SLA is particularly sensitive to light and soil fertility, making it a key discriminator of community adaptation, while LDMC primarily reflects nutrient availability and slower resource turnover (Hodgson et al. 2011; Pietsch et al. 2014; Weemstra et al. 2021). In water‐limited environments, deep roots (greater rooting depth, RRD) enable drought avoidance (Comas et al. 2013), whereas thicker leaves (greater leaf thickness, LT) may serve as a general defence mechanism (Westoby et al. 2002).

Compared to leaf‐level processes, C allocation is treated simplistically in most global ecosystem models (Fatichi et al. 2019). Some (but not all) models invoke aspects of optimal partitioning theory, allowing modelled plants to adjust the fraction of C they allocate to leaves, roots, and stems depending on the relative limitations to plant growth by light, water, and nutrients, particularly nitrogen (N). In these models, root C allocation increases under limited N and water supplies, whereas above‐ground (stem and leaf) C allocation increases under limited light (De Kauwe et al. 2014; Friedlingstein et al. 1999; Krinner et al. 2005; Xia et al. 2017). C allocation models based on the idea of relative resource limitation, such as CASA (Friedlingstein et al. 1999), have been integrated into global land surface models including ISAM, ORCHIDEE, and CTEM (Arora and Boer 2005; Krinner et al. 2005; Song et al. 2013). It should be noted, however, that CASA predicts the fractional allocation of annual biomass production to different pools—whereas the maintenance of plant function requires that relationships among different plant dimensions are conserved, which cannot be achieved using ontogenetically fixed allocation fractions. Other vegetation models, designed for site‐specific application, have been used to test a variety of more complex schemes including (for example) in versions of the CASTANEA (Guillemot et al. 2017), MAIDEN (Gea‐Izquierdo et al. 2015), and PipeQual (Mäkelä et al. 2016) models. Trugman et al. (2019) critically reviewed the various modelling approaches applied to C allocation to date, and noted the potential for optimality‐based schemes to yield more reliable models (with fewer poorly constrained parameters) than many that have been developed. They focused especially on the ratio of leaf to sapwood area as a key quantity that should be predictable based on plant hydraulics, the ratio declining with increasing drought stress. Mäkelä et al. (2008) made a similar argument but focused on N availability, noting that maximization of net primary production requires that plants allocate more to fine roots and less to wood when N is scarce.

Optimal partitioning theory is a special case of the eco‐evolutionary optimality (EEO) approach in plant functional ecology, which has proved powerful in predicting plant responses to environmental conditions (Franklin et al. 2020). “Eco‐evolutionary” refers to the fact that plants adjust their behavior to environmental variables both on ecological time scales through plastic responses (acclimation) and on longer time scales through evolutionary adaptation and environmental selection among taxa (Harrison et al. 2021). An explicit EEO approach has recently been used to account for C allocation to leaves. The basic hypothesis is that C allocation to leaves is limited either by energy supply (in which case, allocation to leaves should maximize net C profit) or by water supply (Zhu et al. 2023). This hypothesis has proved successful in modeling global patterns of the regional and seasonal maximum fractional absorbed photosynthetically active radiation (fAPAR), which depends on leaf area index (LAI) according to Beer's law (Cai et al. 2025).

Here we focus on the root‐shoot ratio (R:S), for which available data are adequate to perform global analyses (Qi et al. 2019). We recognize some key limitations of this metric. In woody plants, R:S unavoidably decreases with size due to the accumulation of stem biomass, and the “root” component includes transport tissues as well as absorptive roots. R:S has nonetheless been shown to correlate with many biotic and abiotic factors at regional and global scales, including light, elevation, mean annual temperature, precipitation, soil texture, soil geochemical properties, land cover, stand condition, and life form (Bukombe et al. 2022; Cairns et al. 1997; Coomes and Grubb 2000; Gerhardt and Fredriksson 1995; Jackson et al. 1996; Luo et al. 2012; Mokany et al. 2006; Peng et al. 2022; Reich et al. 2014; Wang et al. 2008). Empirical studies testing the effect of varying just one or two predictor variables on R:S have obtained contradictory results, however. For example, (Cairns et al. 1997) and (Reich et al. 2014) found no significant correlations between R:S and precipitation, while (Mokany et al. 2006) and (Wang et al. 2008) found that R:S is negatively correlated with water availability. Data‐driven gridded maps of R:S have been generated by training random forest models with multiple climatic, edaphic, and biotic predictors (Guo et al. 2022; Ma et al. 2021). But although this machine‐learning approach provides a powerful short‐cut to prediction, it is hypothesis‐free and has not yielded a clear storyline for the controls of R:S. The combined influence of multiple factors and their relative contributions to R:S remain unclear.

**FIGURE 1:**
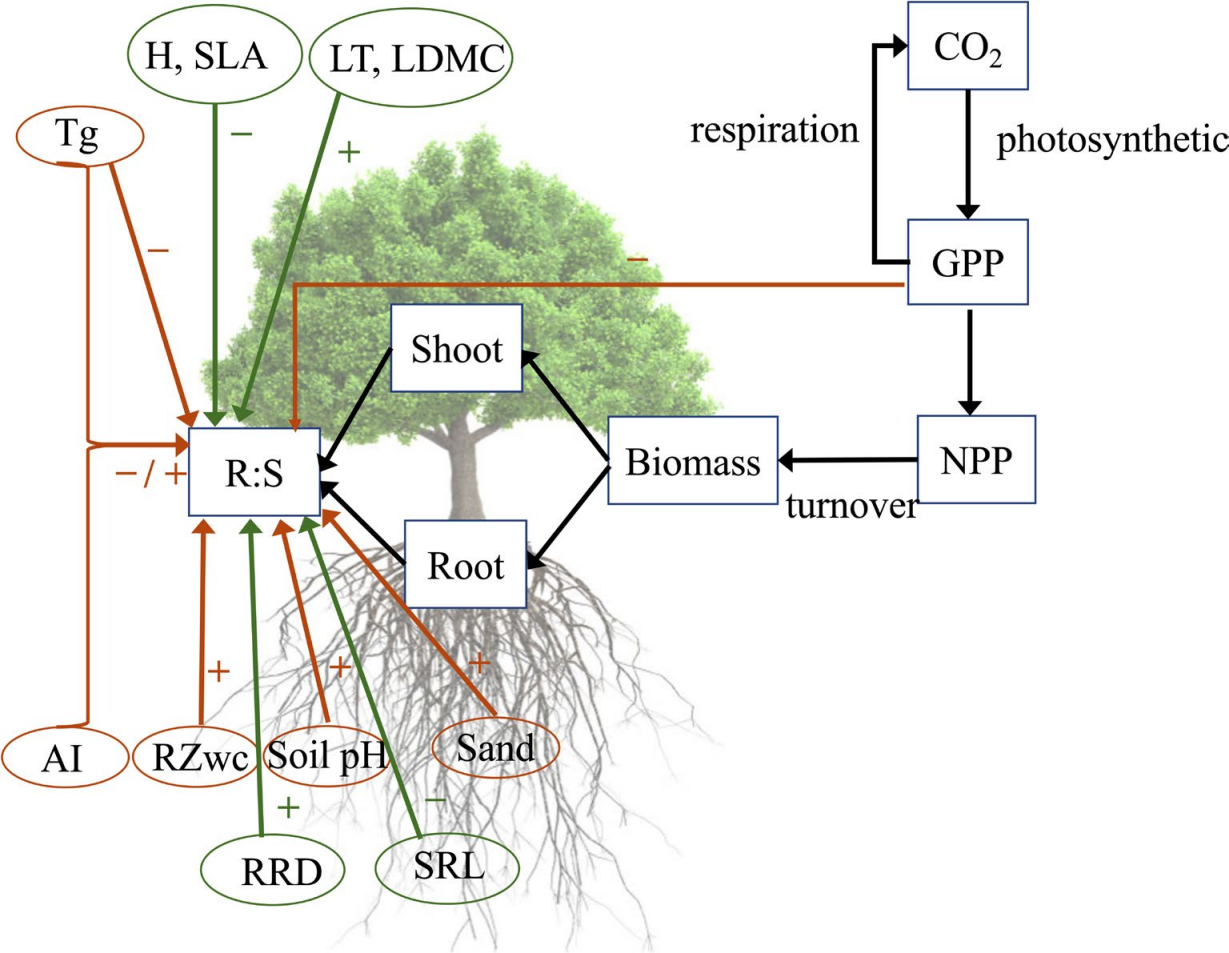
Conceptual framework illustrating how environmental drivers (growing‐season mean temperature, *T*_*g*_; root zone water capacity, RZ_WC_; gross primary production, GPP; soil pH; soil sand content, Sand; aridity index, AI, in tan colour) and plant traits (specific leaf area, SLA; leaf dry matter content, LDMC; leaf thickness, LT; vegetation height, H; root rooting depth, RRD; specific root length, SRL, in green colour) affect root:Shoot ratio (R:S) and biomass allocation strategies.

We assembled a large global set of data, combining several previous compilations, and used it to test EEO‐inspired hypotheses about plant C allocation responses to environmental conditions in woody and herbaceous plants. The environmental controls were expressed in terms of statistical predictors describing aspects of the environment relevant to plant growth. We accordingly used mean growing‐season (rather than mean annual) temperature; gross primary production (GPP); an index of root‐zone water capacity (RZ_WC_) derived from climate properties; soil pH; sand content; and aridity index (AI) as environmental predictors. GPP is accurately simulated by the “P model”, which is itself derived from an optimality‐based theory (Mengoli et al. 2022; Stocker et al. 2020). RZ_WC_ is the maximum amount of water that can be accessed by plants for transpiration (Gao et al. 2014), which is a critical factor in representing the ability of plants to use stored water in soil (Singh et al. 2020). RZ_WC_ can be estimated by maximum cumulative water deficit: the greater RZ_WC_, the longer plants can endure soil moisture limitations. AI is defined as the ratio of annual potential evapotranspiration to annual precipitation and is an indicator of climatological water availability, which has been shown to influence biomass partitioning (Ma et al. 2021). Soil chemical and physical properties (pH and sand content) are major drivers of C allocation due to their effects on nutrient and water storage and uptake (Augusto et al. 2017; Meng et al. 2019) respectively. Soil pH is frequently used as an indicator of various aspects of soil fertility, influencing the availability of major nutrients and thereby influencing many plant traits (Cornwell et al. 2018; Dwyer et al. 2014; Maire et al. 2015; Smith et al. 2019). The trade‐off between root and shoot investment is also linked to plant functional traits (Reich 2014; Westoby et al. 2002; Wright et al. 2001; Wright et al. 2004). We selected six plant and vegetation traits as additional predictors based on their likely relevance for plant C allocation, namely leaf thickness (LT), leaf dry matter content (LDMC), specific leaf area (SLA), rooting depth (RRD), specific root length (SRL), and vegetation height (H). We tested the following specific, EEO‐inspired hypotheses (Figure 1):

- LAI is optimized to the environment (as proposed by Zhu et al. 2023 and Cai et al. 2025), while a certain quantity of roots per unit area is essential to supply a given LAI with water and nutrients. As GPP increases, so does the potential to allocate additional C to stems, providing a competitive advantage in light competition—and leading to a reduction in R:S.
- Higher temperatures lead to faster turnover of nutrients in soils, and roots become more efficient at nutrient uptake due to faster metabolic rates. Higher temperatures therefore lead to a smaller requirement for C allocation to roots (as a given mass of roots can take up more nutrients), and a reduction in R:S. In warm, wet regions, abundant precipitation further promotes aboveground C allocation, decreasing R:S. However, higher temperatures also imply greater evaporative demand and so if water is scarce, this leads to a smaller reduction, or even potentially an increase, in R:S.
- On acid soils, leaves typically have reduced photosynthetic capacity and lower leaf N per unit leaf area (Maire et al. 2015). This implies a lower nutrient uptake requirement from the roots, resulting in a reduction in R:S.
- More C is allocated to roots in climates with seasonal mismatches between water supply and demand, where increased RZ_WC_—maximizing water availability during the dry season—requires an increase in R:S.
- The low water‐holding capacity of sandy soils implies a need for additional C investment in roots for water uptake, and thus an increase in R:S.
- The R:S is hypothesized to increase with traits favoring stress tolerance and deep resource foraging (LT, LDMC, RRD) and to decrease with traits linked to aboveground growth and resource efficiency (H, SLA, SRL).

These hypotheses are not the only ones possible, nor do they uniquely represent EEO strategies. They are, however, the simplest that can be derived from our conceptual model that plants prioritize optimal allocation to foliage plus whatever below‐ground allocation is required to support that foliage.

## 2 Data and Methods

### 2.1 Root:Shoot Biomass Data

**FIGURE 2:**
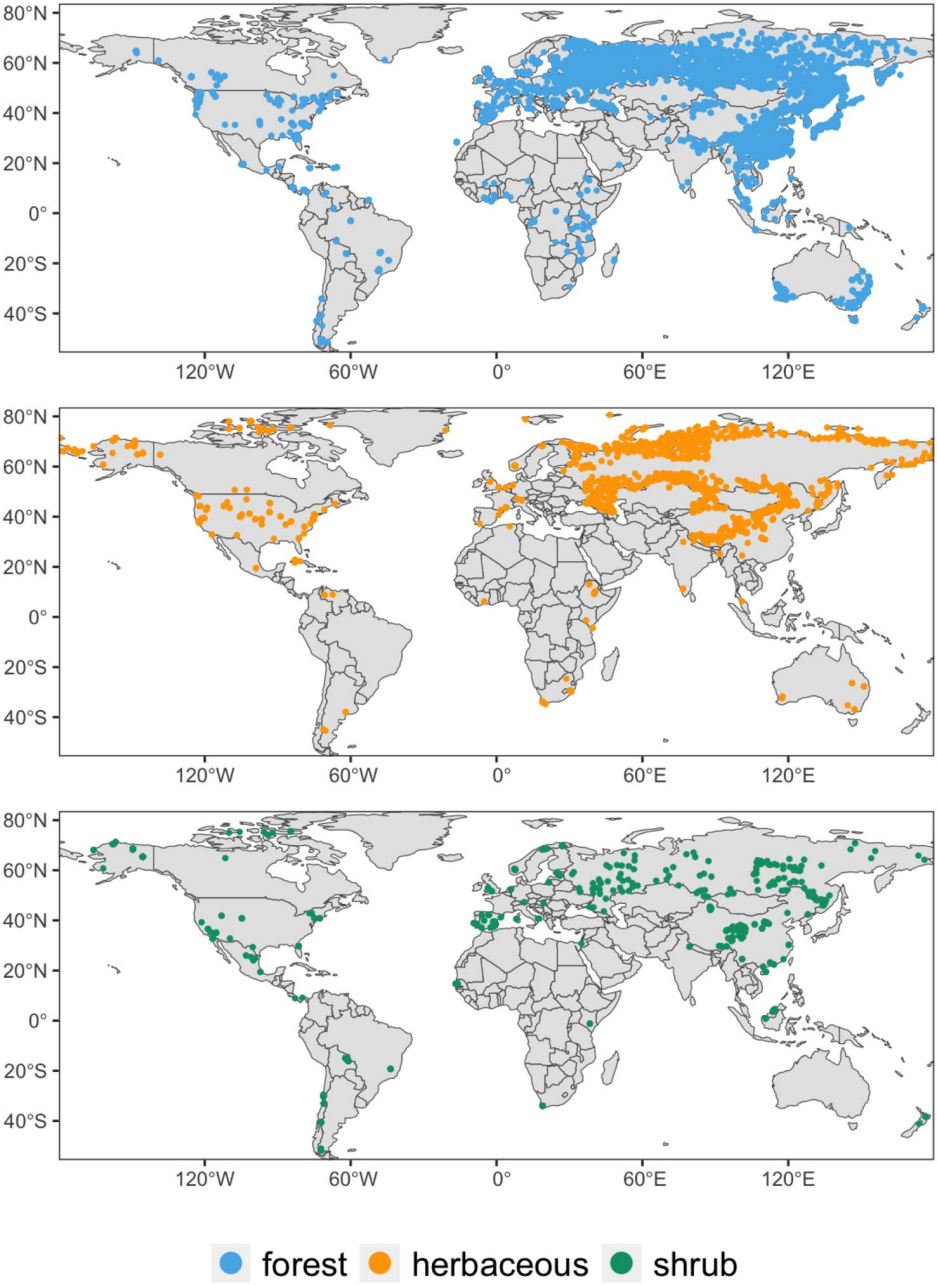
Global root:Shoot ratio (R:S) sample locations. The database of 29,896 R:S records (25,846 forest plants, 2341 herbaceous plants, 1709 shrubs) was aggregated into 13,611 samples with unique locations: forest plants (*n* = 11,372), herbaceous plants (*n* = 1,211), and shrubs (*n* = 1,028). Map lines delineate study areas and do not necessarily depict accepted national boundaries.

Our dataset was assembled from literature containing large compilations of field measurements of R:S in forests, shrublands, and grasslands (Guo et al. 2022; Huang et al. 2021; Ma et al. 2021). Data in Ma et al. (2021) were compiled globally for all three vegetation categories, accounting for 58.7% of the dataset. Data in Huang et al. (2021) and Guo et al. (2022) were derived from forest biomass measurements, accounting for 41.3% of the dataset. Data in Guo et al. (2022) were extracted from forest plots across China (Luo et al. 2014; Wang et al. 2014; Zhang et al. 2016); data in (Huang et al. 2021) were compiled from existing forest biomass databases (Falster et al. 2015; Ledo et al. 2018; Schepaschenko et al. 2017, 2018). In addition to R:S, biomass measurements of root and shoot were included in the data of Huang et al. (2021) and stand age in the data of Guo et al. (2022). Data were selected for analysis according to the following criteria: (1) R:S values were available at both the plot and individual levels; (2) data were from field measurements, i.e., we excluded values derived via allometric equations; (3) both fine and coarse roots were included in belowground biomass; and (4) geographic coordinates were provided. The dataset included 29,896 R:S observations (25,846 forest plants, 2341 herbaceous plants, 1709 shrubs) with georeferenced locations (Figure 2). The data were aggregated into 13,611 samples from forest plants (*n* = 11,372), herbaceous plants (*n* = 1,211), and shrubs (*n* = 1,028), due to a number of samples having identical coordinates. Outliers of R:S within the dataset for each plant type were identified as values falling below the first quartile (Q1) minus 1.5 times the interquartile range (IQR) and values falling above the third quartile (Q3) plus 1.5 times the IQR.

### 2.2 Global Datasets of Environmental and Traits Predictors

We used six environmental predictors, namely mean growing‐season temperature (*T*_*g*_, °C), GPP (gC m^−2^ d^−1^), RZ_WC_ (mm), soil pH, sand content (% by weight), and aridity index (AI). These quantities were chosen due to their relevance to plant biomass allocation and their availability at the global scale (Guo et al. 2022; Huang et al. 2021). Soil pH and sand content data were obtained from the gridded Global Soil Dataset (GSDE). GSDE harmonized the Soil Map of the World with regional and national soil databases and aggregated the data to 30 arc‐second resolution (Wei et al. 2014). We averaged the values of top layers capturing the vertical variation of soil properties to a depth of 0.3 m. For the calculation of AI, potential evapotranspiration was estimated by the SPLASH model (Sandoval et al. 2024), and annual precipitation was obtained as the sum of mean monthly precipitation (mm month^−1^) values extracted from the global CHELSA data set with a spatial resolution of 30 arc‐seconds for 1979–2013 (Karger et al. 2017). Monthly values of *T*_*g*_ were averaged over the thermal growing season, defined as the period with climatological daily mean temperatures > 0°C. Mean daily temperature values were also extracted from the CHELSA data set for 1979–2013 (Karger et al. 2017). Plant‐trait predictors (LT, LDMC, SLA, RRD, SRL, H) were extracted from high‐resolution (1 km) global maps derived by machine learning based on observations from the Global Biodiversity Information Facility, the sPlot database, the TRY plant‐trait database, and global Earth Observation datasets (Lusk et al. 2025).

We used the P model to estimate GPP as a function of environmental conditions. The theory underlying the P model has been described by Prentice et al. (2014) and Wang et al. (2017). Its current standard implementation was described by Stocker et al. (2020). This model combines an optimality‐based theory for the prediction of light use efficiency (LUE) with data on incident photosynthetically active radiation (PAR) and the fraction of absorbed PAR (fAPAR). As inputs for the P model, we used *T*_*g*_, vapour pressure deficit (VPD), PAR, fAPAR, and mean atmospheric pressure calculated by the barometric formula using elevation data extracted from the GTOPO30 digital elevation model. VPD was calculated as the difference between the saturation (*e*_s_) and actual vapour pressures (*e*_a_), where *e*_s_ was calculated from *T*_*g*_ and *e*_a_ derived from relative humidity data in CHELSA. Short‐wave radiation (*R*_SW_) data, used to calculate PAR, were also extracted from CHELSA.

The key equations of the P model are as follows:

(1) GPP=PAR·fAPAR·LUE

(2) $$\mathrm{LUE}=\varphi_{0}\beta \left(\theta \right)\;m^{\prime }\;M_{C},\text{where}\;m^{\prime }=m\surd \left[1-{\left(c^{*}/m\right)}^{2/3}\right]$$

PAR, or more precisely photosynthetic photon flux density (PPFD) (mol m^−2^ d^−1^), was computed from short‐wave radiation (*R*_SW_) as PPFD = 60 60 24 10^−6^ *k*_EC_ *R*_SW_, where *k*_EC_ = 2.04 μmol J^−1^ (Meek et al. 1984). fAPAR data from 2000–2019 at 0.05° spatial resolution (Jiang and Ryu 2016) were derived from the MODIS MOD15A2H LAI/FPAR product (Myneni et al. 2015). *φ*_*0*_ is the intrinsic quantum yield of photosynthesis, accounting for its temperature dependence, and *β(θ)* is an empirical soil moisture stress factor, estimated by the P model using relative soil moisture from the SPLASH model (Sandoval et al. 2024). *M*_C_ is the molar mass of C (12.0107 g mol^−1^), *m* is the factor in the light‐limited assimilation rate function (*ci* – Г*)/(*ci* + 2Г*) where *c*_i_ is the leaf internal CO_2_ partial pressure and Γ* is the photorespiratory compensation point, and c* is a cost factor for maintaining electron transport capacity, estimated as 0.41 based on observed ratios of carboxylation and electron‐transport capacities (Wang et al. 2017). The term *c*_i_ is estimated as an environmentally dependent fraction of the ambient CO_2_ partial pressure, following Prentice et al. (2014).

We used the mass‐curve technique to quantify RZ_WC_ as described in Methods S1. This technique estimates the plant below‐ground soil‐water storage as a cumulative water deficit based on the balance between cumulative inflow and water demand corrected by precipitation seasonality: see Supporting Information, Equation (S1) and Equation (S2). We calculated the cumulative water surplus and deficit for each (monthly) time step using precipitation and evapotranspiration (ET) datasets to compute influxes and effluxes for the 2001–2016 period. The precipitation data were extracted from the WFDE5 dataset derived from the WATCH Forcing Data (WFD) methodology applied to surface meteorological variables from the ERA5 reanalysis (Cucchi et al. 2020). The WFDE5 dataset is at 0.5° grid resolution and has a temporal resolution of 1 hour. The ET data were from MOD16A2, an 8‐day composite product produced at 0.05° resolution.

### 2.3 Statistical Modelling

We fitted parsimonious models using ordinary least squares (OLS) multiple linear regression separately in woody (forest and shrub) and herbaceous species, using the natural logarithm of R:S (equivalent to a logit transformation of the root mass fraction) as the quantity to be predicted. We fitted a linear model of ln R:S with six environmental predictors (*T*_*g*_, GPP, RZ_WC_, soil pH, soil sand content, and AI) and six plant‐trait predictors (LT, LDMC, SLA, RRD, SRL, H), plus the interaction between *T*_*g*_ and AI. This interaction was motivated by a finding by Ma et al. (2021) that the direction of the temperature effect varies according to water availability. Other interactions were not included due to weaker theoretical or empirical support and to avoid overfitting, given the number of predictors already in the model:

(3) $$\begin{array}{c} \ln R:S=\beta_{0}+\beta_{1}T_{g}+\beta_{2}\text{lnGPP}+\beta_{3}\ln{\mathrm{RZ}}_{\mathrm{WC}}\\ \;+\beta_{4}\mathrm{pH}+\beta_{5}\text{Sand}+\beta_{6}\text{lnAI}+\beta_{7}\mathrm{lnH}\\ \;+\beta_{8}\text{lnRRD}+\beta_{9}\text{lnSLA}+\beta_{10}\text{lnSRL}\\ \;+\beta_{11}\text{lnLT}+\beta_{12}\text{lnLDMC}+\beta_{13}T_{g}*\text{lnAI}+\varepsilon \end{array}$$

where *β*_*1*_–*β*_*13*_ are the estimated sensitivities of ln R:S to *T*_*g*_, ln GPP, ln RZ_WC_, soil pH and sand, ln AI, ln H, ln RRD, ln SLA, ln SRL, ln LT, ln LDMC and *T*_*g*_ * ln AI, and *ε* is the residual term. The models were obtained by both‐direction stepwise regression, optimizing the model by adding significant variables and removing insignificant variables to produce the largest increase in R^2^ at each step. We also fitted models for forest and shrubs separately that allow the responses of ln R:S to all of the predictors to differ among forest plants, shrubs and herbaceous plants. We also extended our regression models by including potential natural vegetation (PNV) (Hengl et al. 2018) as a categorical predictor: see Supporting Information.

Regression coefficients were estimated using linear regression (lm function in R, R Core Team 2020). Because of the highly uneven sampling intensity (Figure 2), weights were assigned to samples in inverse proportion to their spatial frequency. Spatial frequency was calculated by the number of neighbouring samples within a distance of 1 km. These weights were applied during the fitting process by the lm function. The data on GPP, RZ_WC_, AI, LT, LDMC, RRD, H, SLA, and SRL were natural log‐transformed due to their highly skewed distributions (Figure S1). Regression relationships were visualized using partial residual plots generated by the visreg package in R (Breheny and Burchett 2017). Partial residual plots display the relationship between the response variable and each predictor variable in turn, using the fitted model to set all other predictors constant at their median values.

Principal component analysis (PCA) was used to identify the environmental predictors causing most of the variance in R:S. The Kaiser–Meyer–Olkin (KMO) statistic was used to determine the effectiveness of dimension reduction by PCA. This statistic ranges from 0 to 1, with higher values indicating greater commonality of variance among variables. All data analysis and graphics were conducted in R v.4.0.5 (R Core Team 2020).

## 3 Results

R:S ranged from 0.009 (woody) to 14.469 (herbaceous), with a median of 0.26 (*n* = 13,611) across all species. The range was from 0.009 to 2.126 in woody plants and from 0.032 to 14.469 in herbaceous plants. The median value for herbaceous plants (4.7) was an order of magnitude higher than that for woody plants (0.25). The highest density of samples occurred at 27.8°C for *T*_*g*_, 159.3 mm for RZ_WC_, 3.46 gC m^−2^ d^−1^ for GPP, 4.1 for soil pH, 78.3% for soil sand content, 1.1 for AI, 0.9 m for H, 14.1 m^2^ kg^−1^ for SLA, 1380.2 cm g for SRL, 0.4 m for RRD, 0.2 mm for LT, and 0.1 g g^−1^ for LDMC (Figure S1). The minimum value of R:S (0.009 in woody) occurred at 18.7°C for *T*_*g*_, 1510.4 mm for RZ_WC_, 4.6 gC m^−2^ d^−1^ for GPP, 4.9 for soil pH, 76.0% for soil sand content, 2 for AI, 1.6 m for H, 10.9 m^2^ kg^−1^ for SLA, 2302.0 cm g for SRL, 0.7 m for RRD, 0.3 mm for LT, and 0.1 g g^−1^ for LDMC. The maximum value of R:S (14.469 in herbaceous) occurred at 19.3°C for *T*_*g*_, 80.3 mm for RZ_WC_, 3.24 gC m^−2^ d^−1^ for GPP, 5.5 for soil pH, 84.0% for soil sand content, 1.2 for AI, 0.7 m for H, 22.8 m^2^ kg^−1^ for SLA, 3235.2 cm g for SRL, 0.4 m for RRD, 0.2 mm for LT, and 0.1 g g^−1^ for LDMC.

**FIGURE 3:**
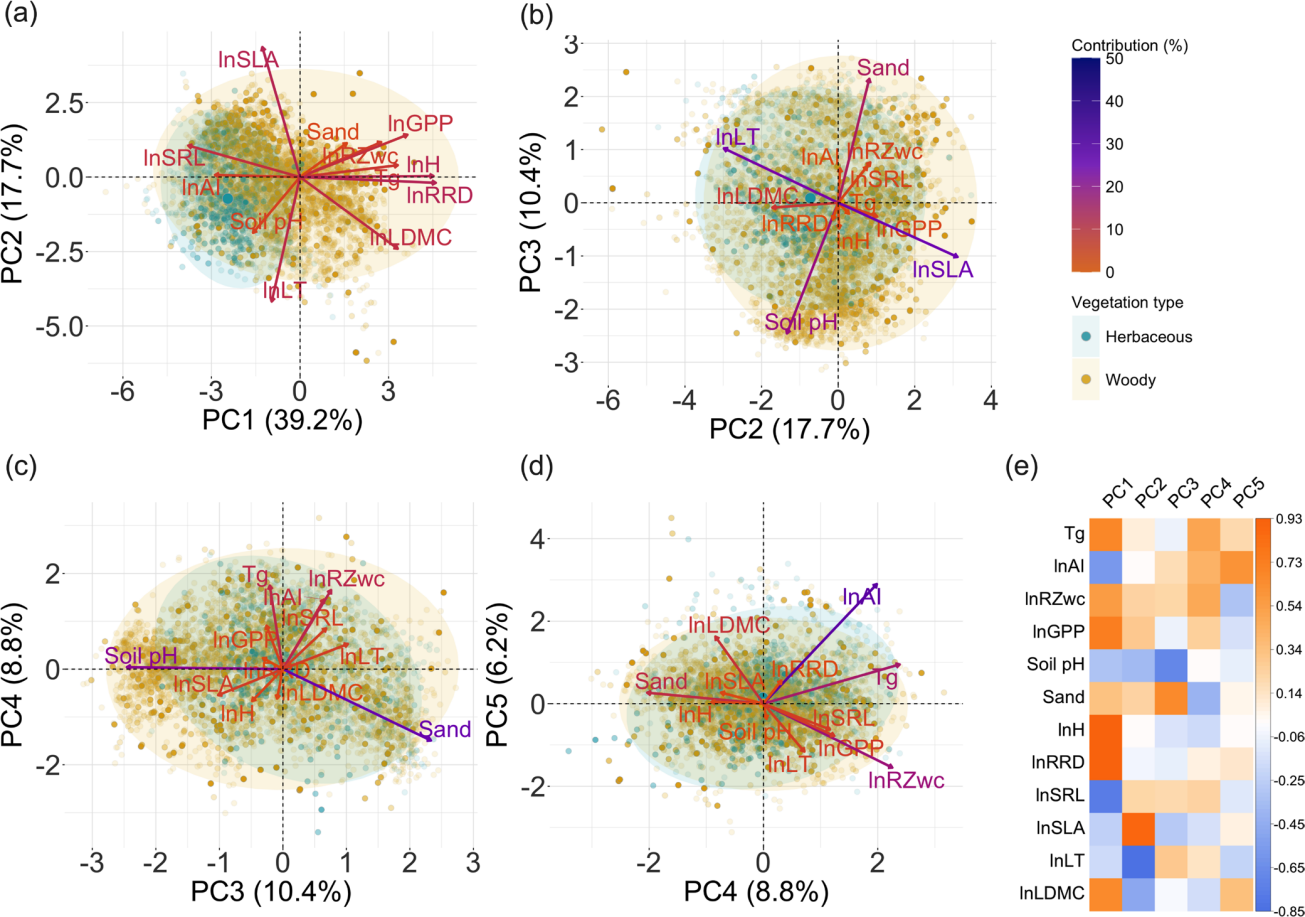
Principal component analysis (PCA) of explanatory variables. (a–d) PCA biplot of observations (yellow points are woody plants and blue points are herbaceous plants) on the climatic, biotic, edaphic predictor and plant traits variables (arrows): Growing‐season mean temperature (*T*_*g*_, °C), natural log‐transformed root zone water capacity (ln RZ_WC_, mm), natural log‐transformed gross primary production (ln GPP, gC m^−2^ d^−1^), soil pH (unitless), soil sand content (Sand, % of weight) and natural log‐transformed aridity index (lnAI, unitless), natural log‐transformed vegetation height (H, m), natural log‐transformed leaf thickness (ln LT, mm), natural log‐transformed leaf dry matter content (ln LDMC, g g^−1^), natural log‐transformed specific leaf area (ln SLA, m^2^ kg^−1^), natural log‐transformed specific root length (ln SRL, cm g^−1^) and natural log‐transformed root rooting depth (ln RRD, m). Panel e represents the correlation of each variable with each axis; coloured arrows represent the contribution of each variable to each axis.

The individual KMO statistics were 0.82 for ln AI, 0.69 for sand content, 0.67 for pH, 0.76 for *T*_*g*_, 0.84 for ln GPP, 0.86 for ln RZ_WC_, 0.79 for ln H, 0.40 for ln SLA, 0.70 for ln SRL, 0.83 for ln RRD, 0.37 for ln LT, and 0.66 for ln LDMC. The overall KMO value was 0.69, greater than the accepted minimum of 0.5. PCA (Figure 3a–e) of the 12 environmental and plant‐trait predictors revealed five independent axes of environmental variation. The first five principal components accounted for 82.4% of total variation; the first two accounted for 56.9% (Figure 3a). The first component was dominated by a positive association with ln GPP, ln RRD, ln H, and a negative association with ln SRL. The second component was dominated by a positive association with ln SLA opposed to ln LT (Figure 3a,b,e). The third component, accounting for 10.4% of the variation, primarily showed a positive contribution of sand content and a negative association with soil pH (Figure 3b,c,e). Some independent variations of *T*_*g*_ and ln RZ_WC_ contributed to the fourth axis, and ln AI and ln LDMC for the fifth axis, accounting for 8.8% and 6.2% of the total variation, respectively (Figure 3d,e).

**FIGURE 4:**
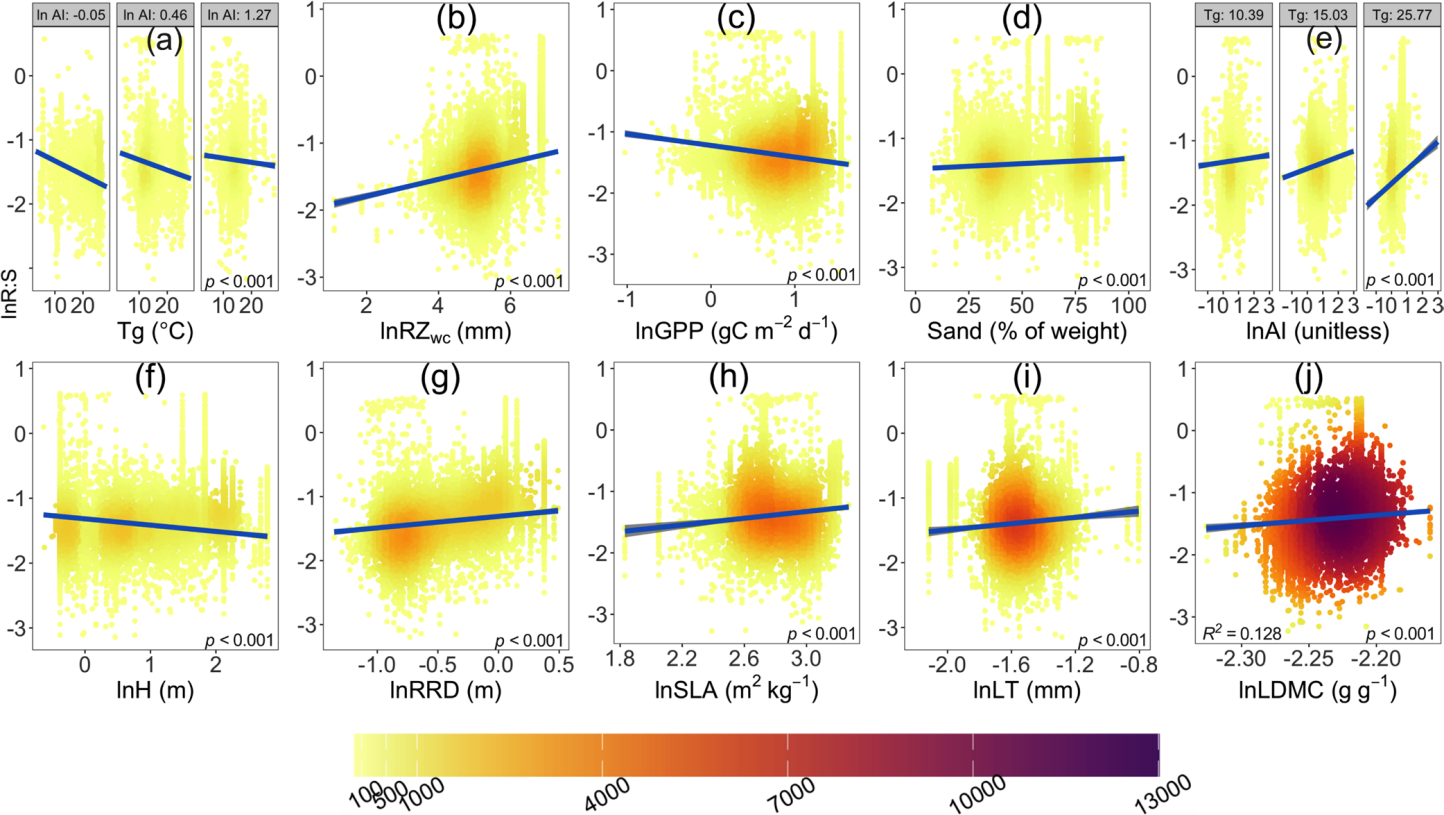
Partial residual plots from the regression of natural log‐transformed root‐shoot biomass ratio (R:S) against explanatory variables in woody species. The plots are from the ordinary least‐squares multiple linear regression in Table 1. (a) Log‐transformed R:S response to growing‐season mean temperature (*T*_*g*_, °C) under different levels of log‐transformed aridity index (ln AI, unitless). (b) Log‐transformed root zone water capacity (ln RZ_WC_, mm). (c) Log‐transformed gross primary production (ln GPP, gC m^−2^ d^−1^). (d) Soil sand content (Sand, % of weight). (e) Log‐transformed aridity index (ln AI). (f) Log‐transformed vegetation height (H, m). (g) Log‐transformed root rooting depth (ln RRD, m). (h) Log‐transformed specific leaf area (ln SLA, m^2^ kg^−1^). (i) Log‐transformed leaf thickness (ln LT, mm). (j) Log‐transformed leaf dry matter content (ln LDMC, g g^−1^). Extreme outliers (0.25% lowest and 0.25% highest) were removed to improve visualization, while all statistical analyses retain the complete dataset. Coefficients and standard errors for the fitted lines are given in Table 1.

**FIGURE 5:**
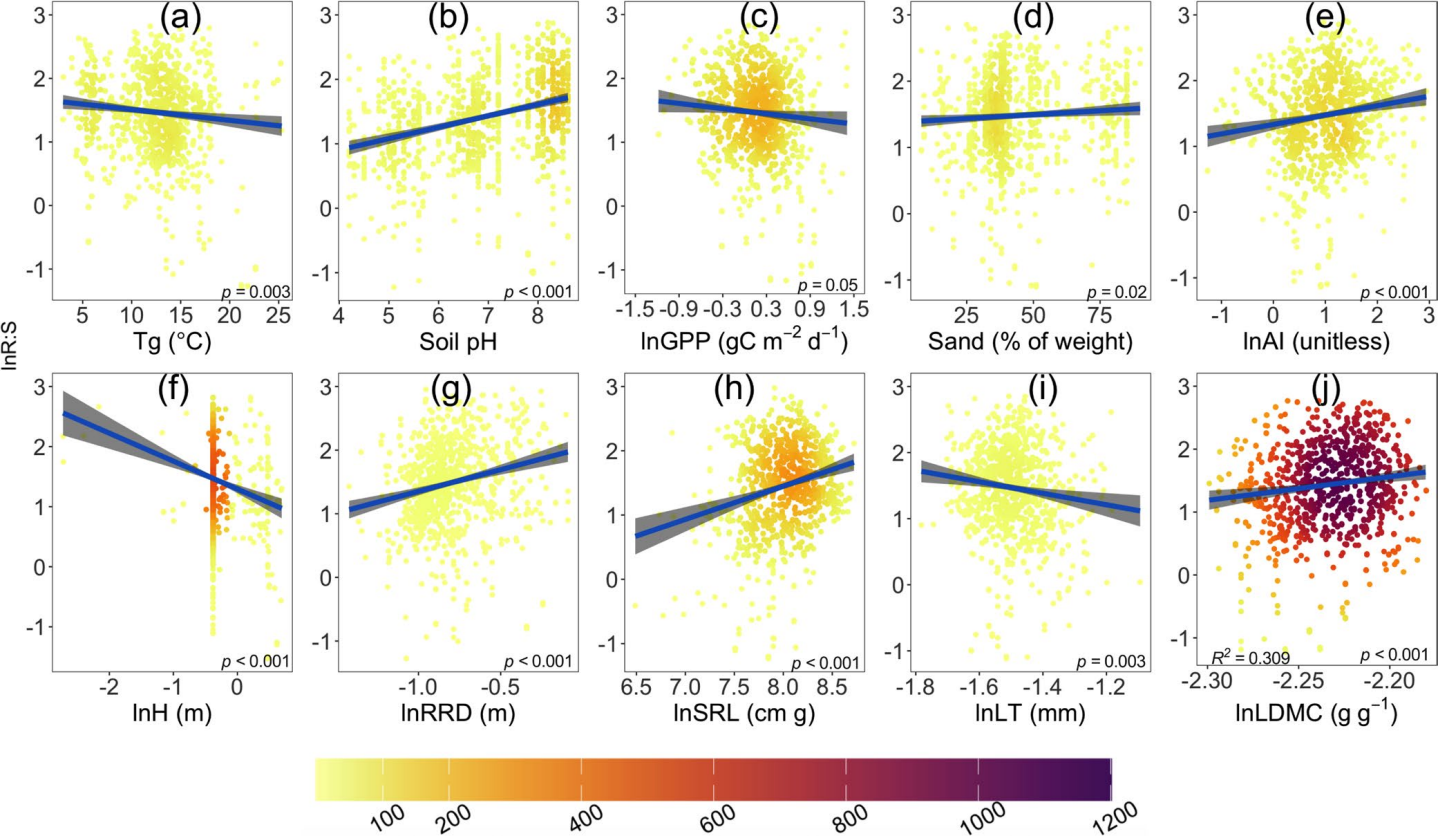
Partial residual plots from the regression of natural log‐transformed root‐shoot biomass ratio (R:S) against explanatory variables in herbaceous species. The plots are from the ordinary least‐squares multiple linear regression in Table 2. (a) Log‐transformed R:S response to growing‐season mean temperature (*T*_*g*_, °C). (b) Soil pH (pH, unitless). (c) Log‐transformed gross primary production (ln GPP, gC m^−2^ d^−1^). (d) Soil sand content (Sand, % of weight). (e) Log‐transformed aridity index (ln AI, unitless). (f) Log‐transformed vegetation height (H, m). (g) Log‐transformed rooting depth (ln RRD, m). (h) Log‐transformed specific root length (ln SRL, cm g^−1^). (i) Log‐transformed leaf thickness (ln LT, mm). (j) Log‐transformed leaf dry matter content (ln LDMC, g g^−1^). Extreme outliers (0.25% lowest and 0.25% highest) were removed to improve visualization, while all statistical analyses retain the complete dataset. Coefficients and standard errors for the fitted lines are given in Table 2.

**FIGURE 6:**
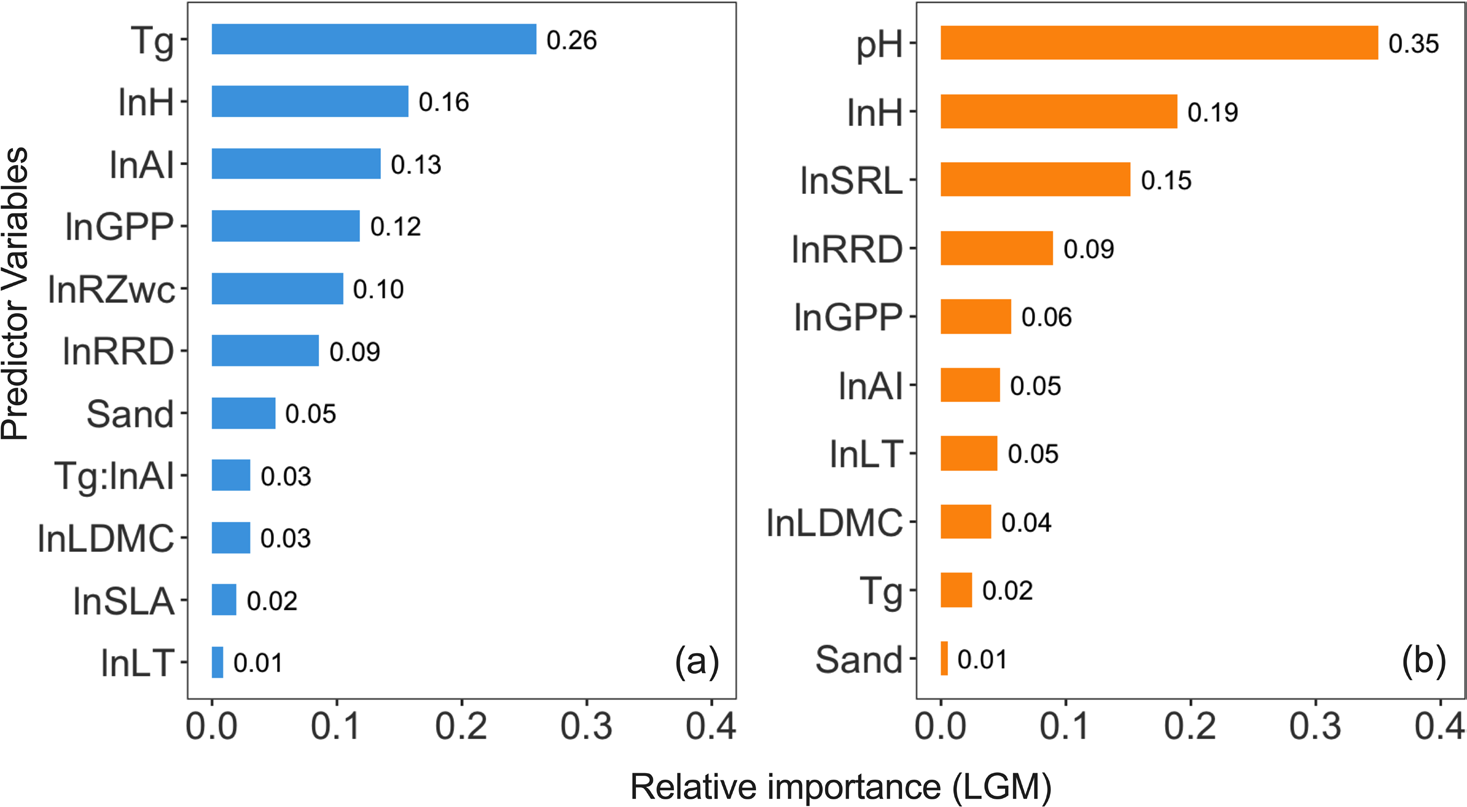
Importance of climate and traits explanatory variables from regression models in woody and herbaceous species. (a‐b) Predictive relative importance for woody (a) and herbaceous (b). Numbers represent relative importance as indicated by the Lindeman–Merenda–Gold (LMG) statistic.

The models for woody and herbaceous plants showed broadly similar patterns in R:S allocation. In both plant types, sand content, ln RRD, and ln LDMC positively influenced R:S, while *T*_g_, ln GPP, and ln H had negative effects (Tables 1, 2, and Figures 4, 5). ln AI negatively and ln RZ_WC_, ln SLA, and ln LT positively affected R:S in woody plants (Table 1, Figure 4b,e,h–i). The partial residual plot of ln AI (Figure 4e) shows a positive trend, due to the modifying effect of the interaction term, whereby the effect of ln AI on the response variable becomes increasingly positive at higher *T*_g_. The interaction term *T*_*g*_ * ln AI was positive (Table 1, Figure 4a), implying that the response of R:S to temperature becomes less negative with increasing climatic dryness in woody plants. In contrast, the herbaceous model showed weaker temperature effects, no interaction of temperature with ln AI, and stronger negative responses to ln LT (Table 2, Figure 5i). R:S in herbaceous plants was also positively associated with soil pH and ln SRL, factors that were not significant in the woody model (Table 2, Figure 5b,h). The herbaceous model yielded higher predictive power (R^2^ = 0.309) than the woody model (R^2^ = 0.128) (Tables 1, 2), reflecting greater trait‐based variability in herbaceous species (Tables 1, 2). The relative importance of predictors suggests that traits play a more significant role than climate variables in explaining biomass allocation in herbaceous plants (Figure 6), highlighting distinct resource‐use strategies between the two plant types.

**TABLE 1:** Summary statistics for the regression of natural log‐transformed root: Shoot biomass ratio (R:S) on predictor variables in woody species. Environmental factors are growing‐season mean temperature (*T*_*g*_, °C), natural log‐transformed root zone water capacity (ln RZ_WC_, mm), natural log‐transformed gross primary production (ln GPP, gC m^−2^ d^−1^), soil sand content (Sand, % of weight), log‐transformed aridity index (ln AI, unitless), and the interaction of *T*_*g*_ and ln AI (*T*_*g*_ * ln AI). Plant traits predictors are log‐transformed vegetation height (H, m), log‐transformed leaf thickness (ln LT, mm), log‐transformed leaf dry matter content (ln LDMC, g g^−1^), log‐transformed specific leaf area (ln SLA, m^2^ kg^−1^), and log‐transformed root rooting depth (ln RRD, m). The standardized coefficient (std. coef.) represents the effect size in standard deviation units.

Woody							
Predictor variable	Coefficient	std.coef.	SE	*t*	*p*	*R* ^*2*^	df
Intercept	1.832	/	0.672	2.728	0.006		
*T*_*g*_ (°C)	−0.021	−0.239	0.001	−14.859	< 0.001		
ln RZ_WC_ (mm)	0.125	0.189	0.008	16.437	< 0.001		
ln GPP (gC/m^2^/d)	−0.190	−0.138	0.016	−11.981	< 0.001		
Sand (% of weight)	0.002	0.066	0.000	7.594	< 0.001		
ln AI (unitless)	−0.085	−0.098	0.025	−3.375	< 0.001	0.128	12,388
ln H (m)	−0.097	−0.176	0.011	−8.236	< 0.001		
ln RRD (m)	0.180	0.154	0.026	6.947	< 0.001		
ln SLA (m^2^ kg^−1^)	0.273	0.109	0.051	5.299	< 0.001		
ln LT (mm)	0.242	0.082	0.061	3.949	< 0.001		
ln LDMC (g g^−1^)	1.679	0.079	0.321	5.228	< 0.001		
*T*_*g*_ * ln AI	0.012	0.009	0.002	7.304	< 0.001		

**TABLE 2:** Summary statistics for the regression of natural log‐transformed root:shoot biomass ratio (R:S) on predictor variables in herbaceous species. Environmental factors are growing‐season mean temperature (*T*_*g*_, °C), natural log‐transformed gross primary production (ln GPP, gC m^−2^ d^−1^), soil pH (unitless), soil sand content (Sand, % of weight), log‐transformed aridity index (ln AI, unitless). Plant traits predictors are log‐transformed vegetation height (ln H, m), log‐transformed leaf thickness (ln LT, mm), log‐transformed leaf dry matter content (ln LDMC, g g^−1^), log‐transformed specific root length (ln SRL, cm g^−1^), and log‐transformed root rooting depth (ln RRD, m). The standardized coefficient (std. coef.) represents the effect size in standard deviation units.

Herbaceous							
Predictor variable	Coefficient	std.coef	SE	*t*	*p*	*R* ^*2*^	df
Intercept	3.470	/	2.299	1.509	0.131		
*T*_*g*_ (°C)	−0.017	−0.092	0.006	−3.024	0.003		
ln GPP (gC/m^2^/d)	−0.135	−0.070	0.069	−1.950	0.051		
Soil pH (unitless)	0.177	0.272	0.018	9.763	< 0.001		
Sand (% of weight)	0.002	0.053	0.001	2.268	0.023		
ln AI (unitless)	0.143	0.128	0.034	4.268	< 0.001	0.309	1200
ln H (m)	−0.465	−0.149	0.078	−5.909	< 0.001		
ln RRD (m)	0.683	0.185	0.108	6.337	< 0.001		
ln SRL (cm g^−1^)	0.516	0.185	0.092	5.616	< 0.001		
ln LT (mm)	−0.870	−0.095	0.289	−3.008	0.003		
ln LDMC (g g^−1^)	3.749	0.106	1.090	3.439	< 0.001		

Separate models for shrubs versus forests revealed some plant‐type specific distinctions in R:S allocation. In forests, *T*_g_, ln AI, pH, ln GPP, and ln SRL negatively influenced R:S, while sand content, ln RZ_WC_, and leaf traits (ln SLA and ln LDMC) had positive effects (Table S1 and Figure S2b–i). Notably, the interaction between *T*_*g*_ and ln AI was positive, showing that the negative effect of temperature on R:S diminishes under drier conditions (Table S1 and Figure S2a). This interaction also caused a positive trend of ln AI in the partial residual plot (Figure S2f). The shrub model showed different trait associations. R:S in shrubs was negatively influenced by plant height (ln H) and root traits (ln RRD and ln SRL), and positively associated with ln GPP and ln LT. ln GPP was included in the forest model, but the other traits (ln H, ln RRD, and ln LT) were not significant. Sand content, ln SLA, and ln LDMC increased, and ln SRL decreased in both forest and shrub models (Table S1 and Figure S2,3). The explanatory power of the shrub model was primarily driven by greater trait‐based variability rather than environmental factors (Table S1 and Figure S4). Compared to the woody and herbaceous models, which share eight overlapping variables (Tables 1, 2), the shrub, forest, and herbaceous models have only four overlapping variables (Table S1). This limited overlap complicates the analysis, and the shrub model showed unexpected patterns, such as negative responses in root traits (ln RRD and ln SRL) and a positive association with ln GPP, inconsistent with our hypotheses. The extended model including PNV provided limited improvement in model fit for both woody and herbaceous vegetation, with R^2^ increased to 15% and 37%, respectively. The inclusion of PNV did not substantially change the significance or direction of the environmental and trait predictors (Table S2).

The variance inflation factors (VIFs) for 12 predictors in both regression models were moderate, ranging from 1.14 – 5.70 (Figure S5), indicating that multicollinearity among these predictors is not a major issue. High VIFs shown for AI and its interaction with *T*_*g*_ (Figure S5) reflect their inevitable intercorrelation. Pairwise correlations between *T*_*g*_, GPP, RZ_WC_, soil pH, soil sand content, ln AI and plant traits variables ranged between −0.79 and 0.86 (Figure S6). The highest correlations were observed among plant traits, e.g., between ln RRD and ln H, and between ln SRL and ln H. Predicted ln R:S based on predictors in woody and herbaceous models was consistent with the observations (Figures S7 and S8) with a coefficient of determination (*R* ^*2*^) between predicted and observed ln R:S of 0.128 and a root mean squared error (RMSE) of 0.52 (Figure S7) in woody plants, and an *R* ^*2*^ of 0.309 and an RMSE of 0.74 in herbaceous plants (Figure S8).

## 4 Discussion

### 4.1 Factors Influencing Root–Shoot Biomass Allocation

The inferred R:S responses to GPP, *T*_*g*_, pH, RZ_WC_, sand content, AI, and plant traits (Table 1) support each of our initial, EEO‐inspired hypotheses. As predicted, our analyses indicated that R:S is negatively related to GPP, *T*_*g*_ (with the relationship to *T*_g_ becoming less negative under dry conditions), and H, while being positively related to RZ_WC_, LT (in woody plants), and to soil pH (in herbaceous plants) and sand content, LDMC, RRD in both plant types. The models explained 13% of the variance in woody plants and 31% in herbaceous plants. The large fraction of variance that remains unexplained by these models is presumably linked to variables that were not recorded, such as forest age and canopy position. R:S tends to decrease with forest stand age, particularly in planted forests, where younger stands experience more intense competition, leading to greater C allocation to roots to enhance water and nutrient uptake (Aakala et al. 2013; Mokany et al. 2006). Additionally, trees in open‐canopy ecosystems show higher R:S ratios compared to those in closed‐canopy areas. This variation is likely a response to differences in light availability and soil water content between the two environments (Durigan et al. 2012).

GPP is the driver of land‐atmosphere carbon dioxide exchange and the largest flux in the global C cycle (Beer et al. 2010). It depends non‐linearly on LAI (Zhao et al. 2021), which controls fAPAR, a key determinant of GPP (Propastin et al. 2012). If maximum LAI is optimized to the environment (Cai et al. 2025) and a certain allocation to roots is essential to maintain the functioning of the leaves (Givnish 1988), then it follows that as GPP increases beyond that required to support maximum LAI (including below‐ground allocation), the excess C is allocated to stems allowing trees to grow and leading to a progressively reduced R:S as GPP increases.

FACE (Free‐Air CO2 Enrichment) experiments have shown that elevated CO_2_ (eCO_2_) leads to higher GPP. However, the allocation of the additional C can vary depending on environmental conditions. In some circumstances (low‐nutrient environments), the additional C goes to roots and has little effect on the woody biomass (Jiang et al. 2020; Weng et al. 2019). This does not conflict with the EEO theory, as eCO_2_ shifts the balance towards increased growth limitation by nutrients.

Photosynthesis and maintenance respiration are linked to tissue N, and C and N allocation are regulated to maximize net growth (Walker et al. 2014). If N uptake is controlled by soil N availability and root exploration for soil N, then from a C budget perspective, enhanced allocation to mycorrhizae and root exudation per fine‐root C at eCO_2_ would constitute a part of NPP that is not accounted for in the R:S. This potential error may have consequences for the soil C budget, but it has no impact on plant growth and N uptake (Franklin et al. 2009).

The inferred negative relationship between R:S and growth temperature under moist conditions is consistent with some previous studies (Luo et al. 2012; Reich et al. 2014). Forests in cold climates assign a lower fraction of biomass to foliage; in particular, evergreen gymnosperms have a low rate of foliage turnover, so less allocation of new biomass is needed to maintain a given foliage mass fraction (Reich et al. 2014). In addition, higher temperatures allow faster nutrient turnover in soil, implying a smaller requirement for C allocation to roots (Mokany et al. 2006). Low temperatures inhibit many aspects of plant function (including photosynthesis, nutrient uptake, and growth) and reduce the root metabolic rate, creating a need for a greater fraction of biomass to be allocated below ground (Qi et al. 2019). Moreover, both the spatial and the annual variability of wood growth are influenced by low‐temperature stress (Guillemot et al. 2017). Increasing temperature could potentially increase the allocation of net primary production to wood production (Hofhansl et al. 2015). However, we also found that the response of R:S to temperature became less negative in woody plants under more arid conditions, suggesting that enhanced biomass allocation below ground is required when either water or nutrient supplies are limiting.

Soil pH affects root dynamics because acid soils constrain root decomposition by reducing soil microbial activity and litter quality (Wang et al. 2020), which in turn constrains nutrient availability. Higher pH leads to greater availability of nutrients held in soil organic matter and decreases the acquisition cost of N and thus the cost of maintaining photosynthetic capacity (Maire et al. 2015). Leaf nitrogen per area (*N*_area_) can be influenced by soil pH (or fertility) but is not unequivocally linked to soil N availability (Dong et al. 2020; Stocker and Prentice 2024). The metabolic component of leaf N is determined by photosynthetic capacity rather than soil N availability (Peng et al. 2021). Soil pH thus affects the balance between the biochemical and water‐transport costs of photosynthesis, and therefore χ, the ratio of leaf‐internal to ambient CO_2_ (Paillassa et al. 2020; Prentice et al. 2014; Westerband et al. 2023). The least‐cost hypothesis states that stomatal behavior minimizes the costs of maintaining carboxylation capacity (*V*_cmax_) and water transport (Prentice et al. 2014). χ is tightly regulated and depends on environmental conditions, with optimal χ increasing with growing‐season air temperature but decreasing with vapour pressure deficit and elevation above sea level (Lavergne et al. 2020; Prentice et al. 2014; Wang et al. 2017). The value of χ, in turn, determines the *V*_cmax_ required to satisfy the coordination hypothesis (i.e., *V*_cmax_ at growth temperatures acclimates to ensure photosynthesis is, on average, neither limited by light nor by Rubisco activity). High χ on acid soils implies a lower optimal photosynthetic capacity, and therefore also a lower area *N*_area_ (Smith et al. 2019; Westerband et al. 2023) and a reduced requirement for roots to take up nutrients. In addition, studies have consistently shown that root elongation, branching, and biomass tend to decrease as a result of acidification, which has been attributed to the leaching of Ca^2+^ and Mg^2+^ and aluminum (Al^3+^) toxicity (Delhaize and Ryan 1995; Kochian et al. 2005; Vanguelova et al. 2007).

A negative relationship between R:S and water availability has also been shown using various climatic measures, including mean annual precipitation, annual mean soil moisture, and aridity index (Ma et al. 2021; Mokany et al. 2006; Wang et al. 2008). Hydraulic demands are critical in allocation processes, with soil hydrology, which drives the transpiration stream, playing a significant role in determining allocation. Roots are highly responsive to soil moisture profiles, extending down to the groundwater table when it is available. Reduced precipitation initially prompts root growth at the expense of shoots, with roots extending deeper towards more stable soil water sources (Potkay et al. 2021). We propose that plants adjust their root‐accessible water storage based on a cost‐minimization strategy, allocating enough C to roots to support water demand in the canopy (Milly 1994). Our finding of a positive relationship between R:S and RZ_WC_ is consistent with this hypothesis. Higher RZ_WC_ occurs where water supply is seasonally mismatched to water demand by plants. Plants develop root systems through greater C allocation to roots that can achieve sufficient water to deal with droughts with specific return periods (Gao et al. 2014). However, different ecosystems have different strategies to survive drought. Forests with low water stress and high tree cover can facilitate moisture uptake by using shallow roots. Moderately drought‐stressed forests invest in more lateral or deeper roots to create enough RZ_WC_ to buffer the water deficit they experience during the dry season. Highly drought‐stressed forests maximize RZ_WC_ via the growth of either lateral or deep roots while reducing moisture loss by leaf shedding (Singh et al. 2020). In savannas, grasses become dormant during the dry season, and woody plants invest below ground in the cheapest way to allow them to compete with grasses for water (Singh et al. 2020).

Edaphic factors control soil fertility as well as water and nutrient fluxes, which influence chemical soil properties and hydrology (Chadwick and Asner 2016; Xia et al. 2016). Soil sand content is a key property affecting soil water holding capacity and water availability (Zhang et al. 2021). Greater C allocation to roots in sandy soils is to be expected because of their low water holding capacity. Our results indicate that sand content has a significant additional effect beyond that of RZ_WC_.

Studies on evolutionarily stable strategies have explored how plant height, shading interactions influenced by SLA and leaf angle, and trade‐offs in height growth between roots, shoots, stems, and foliage shape these patterns (King 2003; Poorter 2001; Westoby et al. 2002). Taller plants and those with high SLA typically allocate less biomass to roots relative to shoots, as they prioritize aboveground growth to maximize light capture and photosynthesis, and in parallel, the magnitude of resource acquisition needed to support canopy photosynthesis (Reich 2014; Westoby et al. 2002). In contrast, plants with thicker leaves (LT), higher LDMC, and deeper roots often allocate more biomass to roots, as these traits are associated with stress tolerance and efficient resource foraging in resource‐limited environments (Schenk and Jackson 2002; Wright et al. 2001; Wright et al. 2004). In addition, SRL plays a critical role, with high SRL generally associated with lower root biomass allocation due to more efficient nutrient uptake per unit root biomass (Freschet et al. 2021; Reich et al. 1998). We hypothesized that R:S would decrease with specific SLA and SRL, but the opposite patterns observed here likely reflect divergent life‐history strategies between woody and herbaceous plants.

### 4.2 Contrasts Between Woody and Herbaceous Vegetation

R:S ratios showed limited variation within forest types, as well as within shrub and herbaceous vegetation groups, suggesting that within‐group differences are relatively small. However, some herbaceous types display wider R:S distributions than woody vegetation (Figure S10). This suggests that while broad functional groups capture some differences, much of the variation in R:S can be explained by continuous environmental and trait gradients. Our regression results showed that including PNV did not substantially alter the significance or direction of key environmental and trait predictors, and several vegetation categories had non‐significant effects. Our estimates align with those of more complex models—despite the simplicity of our approach—supporting the notion that major model divergences may arise more from internal parameterizations and PFT definitions than from fundamental differences in environmental constraints (Bloom and Williams 2015; Harrison et al. 2021). These findings thus support the use of PFT‐independent trait‐ and environment‐based models to capture variation in C allocation.

Interpretation of differences in the fitted relationships between plant types is hindered by the highly uneven distribution of shrubs in the data set. Shrubs are mainly distributed in the data set in either cold, low‐GPP environments with low RZ_WC_ (tundra) or warm, higher‐GPP environments with high RZ_WC_ (temperate and tropical shrublands), while herbaceous plants are largely confined to cold, low‐GPP environments with low RZ_WC_ (Figure S1). This uneven distribution induced the obscured non‐significant responses of R:S to *T*_*g*_, AI, and RZ_WC_ in shrubs. However, the negative relationship of GPP to R:S was found to be consistent in forest and herbaceous plants. The anomalous increase of R:S with GPP shown by shrubs likely reflects the contrast between tundra with low GPP and warmer, drier shrublands with higher GPP. In forests and herbaceous plants, the negative effect of *T*_*g*_ on R:S was shown here to be most pronounced in moist regions; *T*_*g*_ showed a less negative, or even a positive, effect on R:S in drier regions in forests. This is presumably because higher temperature under dry conditions exacerbates water limitation, leading to increased below‐ground investment (Ma et al. 2021).

The positive effect of pH on R:S is primarily driven by herbaceous plants and is absent in woody plants. The deviations from the hypothesized relationships—the positive effects of SLA on R:S in woody plants and the positive effect of SRL on R:S in herbaceous plants—can be explained by trait‐function relationships and ecological strategies. In herbaceous plants, the combined effects of pH and SRL suggest an adaptation for efficient nutrient acquisition, as higher soil pH enhances nutrient availability, promoting finer, more extensive root systems (high SRL) to maximize uptake efficiency (Freschet et al. 2021). Species with thinner roots and high SRL may be more competitive in coarse‐grained soils by increasing total absorbing length per unit C invested (Holdaway et al. 2011) or dry upland sites by reducing radial resistance to water movement (Comas et al. 2013). In contrast, woody plants prioritize long‐term resource conservation and deeper rooting systems, as their root strategies are shaped more by structural investment and stress tolerance than by rapid nutrient acquisition (Reich 2014). High SLA (indicating resource‐acquisitive leaves) reflects a strategy of efficient light capture and rapid C assimilation, which may simultaneously support greater investment in roots to enhance water and nutrient foraging in competitive or resource‐limited environments (Poorter et al. 2012; Reich 2014; Wright et al. 2004). Unlike herbaceous plants, which focus on rapid growth and reproduction, their resource allocation is more strongly driven by aboveground growth and short‐term nutrient acquisition rather than long‐term resource foraging (Freschet et al. 2021).

## 5 Conclusion

This study has focused on deriving parsimonious statistical models to summarize the environmental and trait‐based factors controlling plant C allocation. Across all climatic regions and plant types, the available data support a series of hypotheses based on EEO theory: higher R:S values are found in colder (for forests) and/or drier ecosystems with higher RZ_WC_, lower GPP, and on sandy and (for herbaceous plants) alkaline soils, which relate to stress‐tolerant traits (LT, LDMC, RRD), while traits linked to aboveground growth and resource efficiency (H, SLA, SRL) reduce R:S. These findings support a general approach to plant C allocation modelling in which the relative allocation to roots is related to the resource uptake requirements of leaves, while C in excess of these requirements is allocated to stems—consistent with an evolutionarily stable strategy (ESS) for plant growth in different environments (Farrior et al. 2013; Mcnickle et al. 2016).

The relatively low explanatory power of our models, particularly for woody plants, suggests that plasticity in allocation strategies and unmeasured sources of variation—such as forest age or canopy position—may influence the extent to which plants can invest surplus C in height growth that enhances light competition after meeting demands for photosynthetic and belowground support. Deviations from these hypothesized relationships underscore the role of trait‐function interactions and highlight the diverse ecological strategies of different plant functional types. These results emphasize the importance of integrating both environmental drivers and plant traits in modeling C allocation across ecosystems and plant types. While there is no universal method for directly connecting data analysis to model development, empirical data play a crucial role in evaluating the performance of models. Our models are based on correlative analyses and large‐scale data can provide support for but not proof of the hypotheses being tested. Recognizing these limitations, our results nonetheless build on EEO to provide robust, rigorous predictions (well supported by empirical data) of how plant allocation varies with environmental conditions.

## Author Contributions

**Ruijie Ding:** conceptualization, data curation, formal analysis, methodology, software, validation, visualization, writing – original draft, writing – review and editing. **Rodolfo L. B. Nóbrega:** methodology, software, writing – review and editing. **Iain Colin Prentice:** conceptualization, funding acquisition, methodology, project administration, supervision, writing – review and editing.

## Conflicts of Interest

The authors declare no conflicts of interest.

## Supporting information


**Data S1:** Supporting Information.

## Data Availability

All data and code used in this study have been made openly available on Zenodo. This includes the observed R:S data compiled from the cited publications, along with the corresponding observed and predicted climate variables required to the analyses and results. The full dataset and code can be accessed at https://doi.org/10.5281/zenodo.15170971. Soil properties were obtained from the Global Soil Dataset for use in Earth System Models (GSDE) at (http://globalchange.bnu.edu.cn/research/soilw). Plant‐trait data were obtained from an integration of GBIF, sPlot, TRY, and Earth observation datasets at (https://geosense‐freiburg.github.io/global‐traits/). Climate data were obtained from CHELSA (https://chelsa‐climate.org/) and the WFDE5 dataset, based on the WATCH Forcing Data methodology applied to ERA5 reanalysis, at (https://doi.org/10.24381/cds.20d54e34). fAPAR were obtained from MODIS MOD15A2H product at (https://www.environment.snu.ac.kr/). The compiled R:S dataset includes field measurements from Ma et al. (2021) (https://doi.org/10.1038/s41559‐021‐01485‐1), Huang et al. (2021) (https://doi.org/10.6084/m9.figshare.12199637.v1), and Guo et al. (2022) (https://doi.org/10.6084/m9.figshare.19397930.v1).
