# Supplementary material for: Global Assessment of Environmental and Plant‐Trait Influences on Root: Shoot Biomass Ratios
Source: Glob Chang Biol. 2025 Oct 7;31(10):e70543. doi: 10.1111/gcb.70543 (PMC12501925; doi:10.1111/gcb.70543)
Supplement: Supplementary file 1 — Data S1: Supporting Information. [file GCB-31-e70543-s001.pdf]

Supplementary Information

Method S1

We used an adapted version of the mass-curve technique (Gao *et al.*, 2014) to quantify root-zone water capacity (RZ_{WC}). This approach is based on the assumption that plant below-ground soil-water storage can be estimated by computing the cumulative water deficit derived from the balance between cumulative vertical water influxes and effluxes. The adapted method assumes that physiologically mature plants will not expand their root systems as the result of a single drought event with a relatively high return period. Instead, it uses the climatological water deficit as a reliable proxy for the RZ_{WC} developed as a result of the long-term natural process by which native plants have become adapted to their habitats. We adjust RZ_{WC} by multiplying it by the precipitation seasonality index (S_i; Walsh & Lawler (1981) and a universally fitted multiplier and exponent, i.e. 0.9 $S_i^{-0.5}$ following Nóbrega et al. (2025).

We calculated the cumulative water surplus and deficit for each (monthly) time step using precipitation as water and evapotranspiration (ET) datasets to compute influxes and effluxes for the 2001–2016 period. The precipitation data were extracted from the WFDE5 dataset derived from the WATCH Forcing Data (WFD) methodology applied to surface meteorological variables from the ERA5 reanalysis (Cucchi et al., 2020). The WFDE5 dataset is at 0.5° grid resolution and has a temporal resolution of one hour. The ET was obtained from the MOD16A2 MODIS product, an 8-day composite product produced at 0.05° resolution. We used bilinear interpolation of the four nearest grid cells using the raster package (Hijmans, 2020) in the R programming language (R Core Team, 2020) to extract data from the datasets.

We verified the RZ_{WC} estimates via a root-zone moisture stress (RZ_{STRESS}) function (Eq S1), which is applied to the gross primary productivity (GPP) outputs of the P-model available at Stocker *et al.* (2020) and compared them to the GPP measurements of 72

sites available in the FLUXNET2015 dataset (Pastorello *et al.*, 2020). We computed S_i based on the annual mean and monthly atmospheric precipitation ($\bar{P}$ and $\overline{x_n}$, Eq S2). The adapted methodology for estimating RZ_{WC} showed better performance than the P-model's default soil moisture function (Fig. S9), supporting the use of this methodology for estimating RZ_{WC}.

$$RZ_{STRESS} = 1 - min\left(1, \frac{0.9S_i^{0.5}\ RZ_{WD}}{RZ_{WC}}\right) \quad \text{(S1)}$$

$$S_i = \frac{1}{\bar{P}} \sum_{n=1}^{n=12} \left| \overline{x_n} - \frac{\bar{P}}{12} \right| \quad \text{(S2)}$$

Table S1. Summary statistics for the regression of natural log-transformed root:shoot biomass ratio (R:S) on predictor variables in forest and shrubs species. Environmental factors are growing-season mean temperature (T_g, ˚C), natural log-transformed root zone water capacity (ln RZ_{WC}, mm), natural log-transformed gross primary production (ln GPP, gC m^{-2} d^{-1}), soil sand content (Sand, % of weight), log-transformed aridity index (ln AI, unitless), and the interaction of T_g and ln AI (T_g * ln AI). Plant traits predictors are log-transformed vegetation height (H, m), log-transformed leaf thickness (ln LT, mm), log-transformed leaf dry matter content (ln LDMC, g g^{-1}), log-transformed specific leaf area (ln SLA, m^2 kg^{-1}), log-transformed specific root length (ln SRL, cm g^{-1}) and log-transformed root rooting depth (ln RRD, m).

Forest							
Predictor variable	Coefficient	std.coef	SE	*t*-value	*p*-value	R^2	df
Intercept	1.270	/	0.503	2.527	0.012		
T_g (˚C)	−0.015	−0.193	0.001	−14.476	< 0.001		
ln RZ_{WC} (mm)	0.077	0.128	0.006	12.401	< 0.001		
ln GPP (gC m^{-2} d^{-1})	−0.140	−0.116	0.011	−12.293	< 0.001		
Soil pH (unitless)	−0.011	−0.038	0.003	−3.690	< 0.001		
Sand (% of weight)	0.001	0.045	0.000	5.239	< 0.001		
ln AI (unitless)	−0.132	−0.184	0.021	−6.363	< 0.001	0.111	11361
ln SRL (cm g)	−0.087	−0.082	0.014	−6.101	< 0.001		
ln SLA (m^2 kg^{-1})	0.279	0.138	0.023	12.114	< 0.001		
ln LDMC (g g^{-1})	1.306	0.075	0.258	5.058	< 0.001		
T_g * ln AI	0.016	0.015	0.001	12.229	< 0.001		

Shrub

Predictor variable	Coefficient	std.coef	SE	*t*-value	*p*-value	R^2	df
Intercept	35.862	/	3.320	10.803	< 0.001		
ln GPP (gC m^{-2} d^{-1})	0.380	0.300	0.077	4.920	< 0.001		
Sand (% of weight)	0.003	0.071	0.001	3.205	0.001		
ln H (m)	−0.560	−0.739	0.076	−7.313	< 0.001	0.290	1019
ln RRD (m)	−1.002	−0.631	0.128	−7.826	< 0.001		
ln SRL (cm g)	−0.844	−0.405	0.107	−7.924	< 0.001		
ln SLA (m^2 kg^{-1})	1.895	0.327	0.336	5.635	< 0.001		
ln LT (mm)	2.472	0.383	0.398	6.207	< 0.001		
ln LDMC (g g^{-1})	14.377	0.391	1.725	8.332	< 0.001		

Table S2. Summary statistics for the regression of natural log-transformed root:shoot biomass ratio (R:S) on predictor variables in woody and herbaceous species. Environmental factors are growing-season mean temperature (T_g, ˚C), natural log-transformed root zone water capacity (ln RZ_{WC}, mm), natural log-transformed gross primary production (ln GPP, gC m^{-2} d^{-1}), soil sand content (Sand, % of weight), log-transformed aridity index (ln AI, unitless), and the interaction of T_g and ln AI (T_g * ln AI). Plant traits predictors are log-transformed vegetation height (H, m), log-transformed leaf thickness (ln LT, mm), log-transformed leaf dry matter content (ln LDMC, g g^{-1}), log-transformed specific leaf area (ln SLA, m^2 kg^{-1}), log-transformed specific root length (ln SRL, cm g^{-1}) and log-transformed root rooting depth (ln RRD, m). Potential Natural Vegetation (PNV) types based on BIOME 6000 classes (20).

Woody							
Predictor	Coefficient	std.coef	SE	*t*-value	*p*-value	R^2	df

variable							
Intercept	1.250	/	0.730	1.731	0.087		
T_g (˚C)	−0.018	−0.205	0.002	−11.106	< 0.001		
ln RZ_{WC} (mm)	0.104	0.156	0.008	13.268	< 0.001		
ln GPP (gC m^{-2} d^{-1})	−0.162	−0.119	0.017	−9.811	< 0.001		
Sand (% of weight)	0.002	0.006	0.000	7.0411	< 0.001		
ln AI (unitless)	−0.079	−0.091	0.026	−3.012	0.003		
ln H (m)	−0.076	−0.138	0.013	−6.028	< 0.001		
ln RRD (m)	0.194	0.167	0.029	6.653	< 0.001		
ln SLA (m^2 kg^{-1})	0.296	0.119	0.054	5.480	< 0.001		
ln LT (mm)	0.211	0.071	0.064	3.309	< 0.001		
ln LDMC (g g^{-1})	1.422	0.067	0.351	4.056	< 0.001		
T_g * ln AI	0.011	0.020	0.002	6.491	< 0.001		
Cold evergreen needleleaf forest	−0.032	/	0.023	−1.395	0.163		
Cold mixed forest	−0.053	−0.043	0.026	−2.069	0.039		
Cool evergreen needleleaf forest	0.101	0.113	0.041	0.251	0.802		
Cool temperate rainforest	−0.135	−0.156	0.059	−2.303	0.021	0.152	12365
Dessert	−0.145	−0.219	0.046	−3.174	0.002		
Erect dwarf shrub tundra	−0.090	−0.066	0.042	−2.158	0.031		

Graminoid and forb tundra	0.266	10.698	0.032	8.423	< 0.001
Low and high shrub tundra	−0.100	−0.182	0.051	−1.945	0.052
Prostrate dwarf shrub tundra	−0.048	−0.041	0.052	−0.929	0.353
Steppe	−0.077	−0.031	0.028	−2.785	0.005
Temperate deciduous broadleaf forest	−0.066	−0.022	0.028	−2.366	0.018
Temperate evergreen needleleaf open woodland	0.001	0.001	0.072	0.014	0.989
Temperate sclerophyll woodland and shrubland	−0.311	/	0.069	−4.501	< 0.001
Tropical deciduous broadleaf forest and woodland	−0.089	−0.007	0.048	−1.852	0.064
Tropical evergreen broadleaf forest	−0.129	−1.440	0.055	−2.339	0.019
Tropical savanna	0.201	0.231	0.061	3.283	0.001
Tropical semi evergreen broadleaf forest	−0.413	−0.622	0.061	−6.709	< 0.001

Warm temperate evergreen and mixed forest	−0.115	−0.084	0.032	−3.633	< 0.001		
Xerophytic woods scrub	0.497	20.007	0.081	6.140	< 0.001		

Herbaceous

Predictor variable	Coefficient	std.coef	SE	*t*-value	*p*-value	R^2	df
Intercept	3.399	/	2.354	1.444	0.149		
T_g (˚C)	−0.014	−0.074	0.006	−2.406	0.016		
ln GPP (gC m^{-2} d^{-1})	−0.158	−0.082	0.075	−2.106	0.035		
Soil pH (unitless)	0.092	0.141	0.022	4.174	< 0.001		
Sand (% of weight)	0.001	0.034	0.001	1.460	0.144		
ln AI (unitless)	0.209	0.187	0.035	6.052	< 0.001		
ln H (m)	−0.353	−0.113	0.081	−4.374	< 0.001		
ln RRD (m)	0.552	0.150	0.123	4.488	< 0.001		
ln SRL (cm g^{-1})	0.362	0.130	0.094	3.868	< 0.001		
ln LT (mm)	−1.035	−0.112	0.293	−3.868	< 0.001		
ln LDMC (g g^{-1})	3.194	0.090	0.293	2.844	0.005		
Cold evergreen needleleaf forest	−0.078	/	0.103	−0.764	0.445	0.366	1183
Cold mixed forest	0.449	0.185	0.112	4.002	< 0.001		

Cool evergreen needleleaf forest	0.307	1.655	0.198	1.547	0.122
Cool temperate rainforest	0.997	0.892	0.367	2.716	0.007
Desert	0.403	0.210	0.125	3.219	0.001
Erect dwarf shrub tundra	0.438	0.673	0.180	2.440	0.015
Graminoid and forb tundra	0.632	14.469	0.124	5.115	< 0.001
Low and high shrub tundra	0.430	0.138	0.224	1.922	0.055
Prostrate dwarf shrub tundra	0.616	0.167	0.240	2.566	0.010
Steppe	0.257	0.092	0.103	2.496	0.013
Temperate deciduous broadleaf forest	−0.006	−0.001	0.179	0.061	0.952
Temperate evergreen needleleaf open woodland	0.011	0.0003	0.179	−0.032	0.974
Tropical semi evergreen broadleaf forest	0.676	/	0.509	1.328	0.185
Warm temperate evergreen and mixed forest	0.688	0.283	0.371	1.854	0.064

Xerophytic woods scrub	−0.501	−2.706	0.317	−1.582	0.114

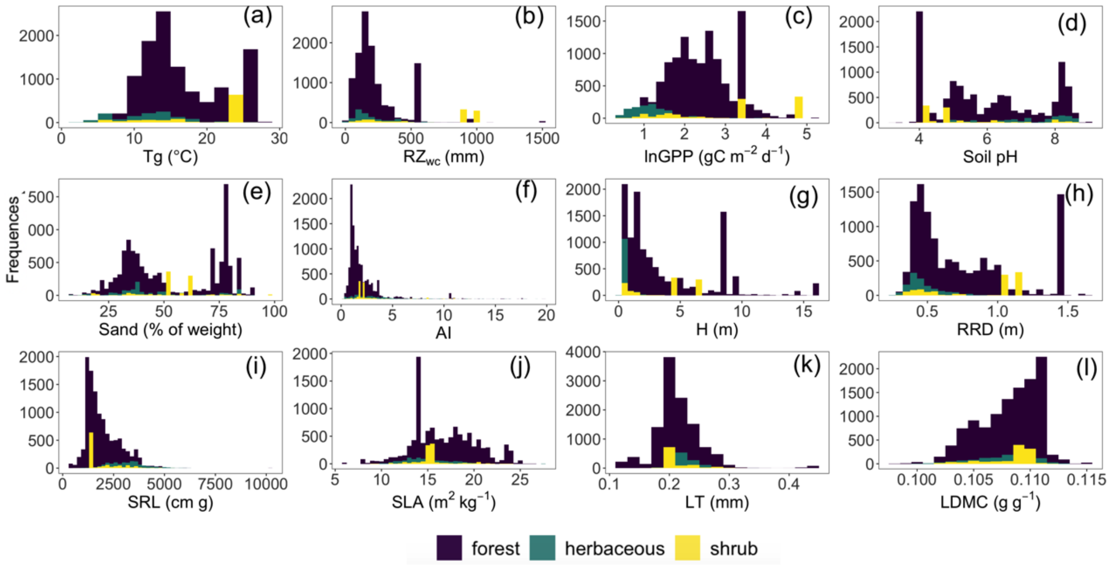

Fig. S1 Distributions of climatic and traits variables in the dataset. a: growing-season mean temperature (T_g, ˚C). b: root zone water capacity (RZ_{WC}, mm).c: gross primary production (GPP, gC m^{-2} d^{-1}). d: soil pH (unitless). e: soil sand content (Sand, % of weight). f: aridity index (AI, unitless). g: vegetation height (H, m). h: root rooting depth (RRD, m). i: specific root length (SRL, cm g^{-1}). j: specific leaf area (SLA, m^2kg^{-1}). k: leaf thickness (LT, mm). l: leaf dry matter content (LDMC, g g^{-1}). Different colours in the histogram represent vegetation types (dark purple for forest plants, green for herbaceous plants, and yellow for shrubs).

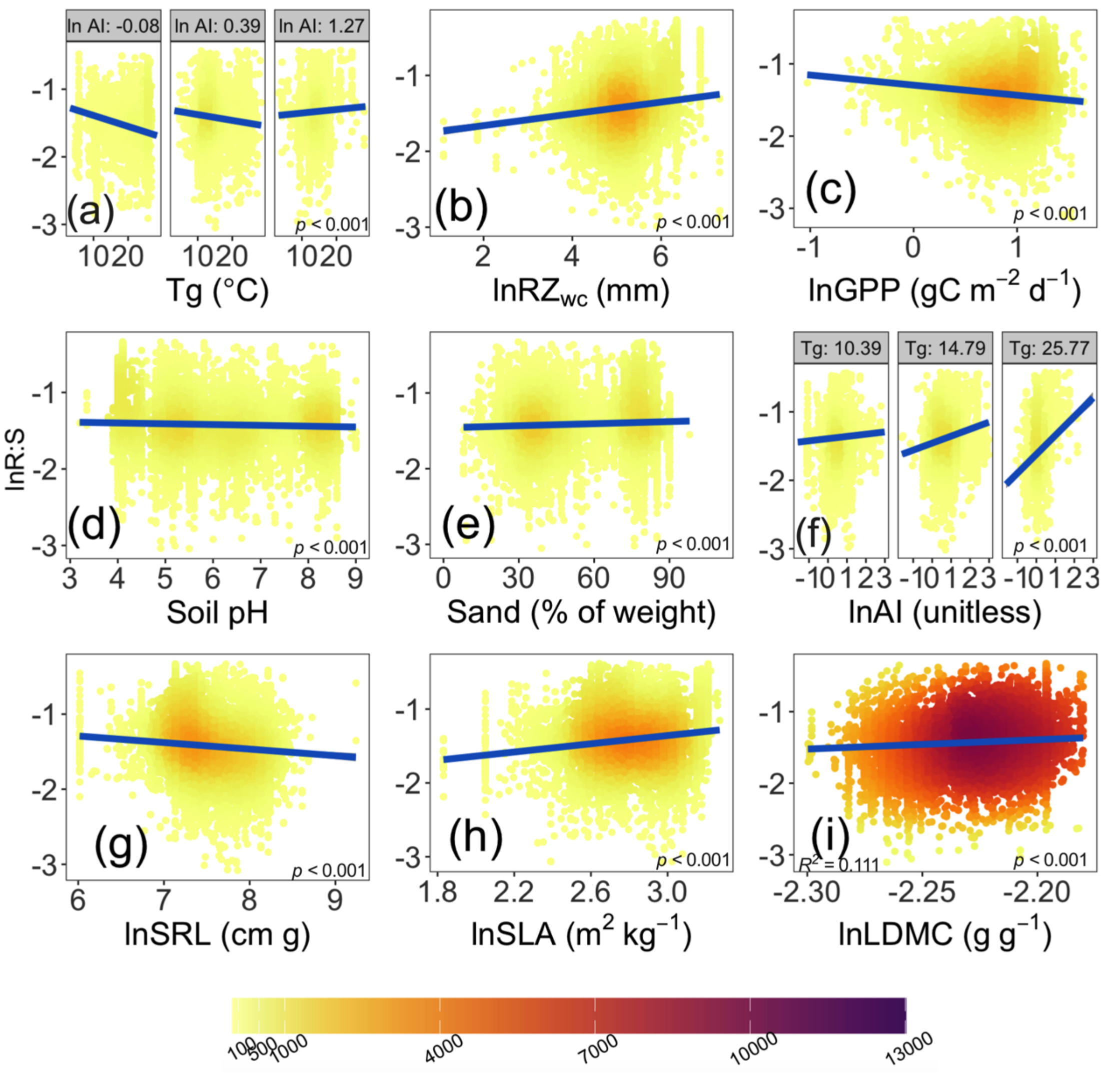

Fig. S2 Partial residual plots from the regression of natural log-transformed root-shoot biomass ratio (R:S) against explanatory variables in forest species. The plots are from the ordinary least-squares multiple linear regression in Table S1. a: log-transformed R:S response to growing-season mean temperature (T_g, ˚C) under different levels of log-transformed aridity index (ln AI). b: log-transformed root zone water capacity (ln RZ_{WC}, mm). c: log-transformed gross primary production (ln GPP, gC m^{-2} d^{-1}). d: soil pH (pH, unitless). e: soil sand content (Sand, % of weight). f: log-transformed aridity index (ln AI, unitless). g: log-transformed specific root length (ln SRL, cm g). h: log-transformed specific leaf area (ln SLA, m^2 kg^{-1}). i: log-transformed leaf dry matter content (ln LDMC, g g^{-1}). Extreme outliers (0.25% lowest and 0.25% highest) were removed to improve visualization, while all statistical analyses retain the

complete dataset. Coefficients and standard errors for the fitted lines are given in Table S1.

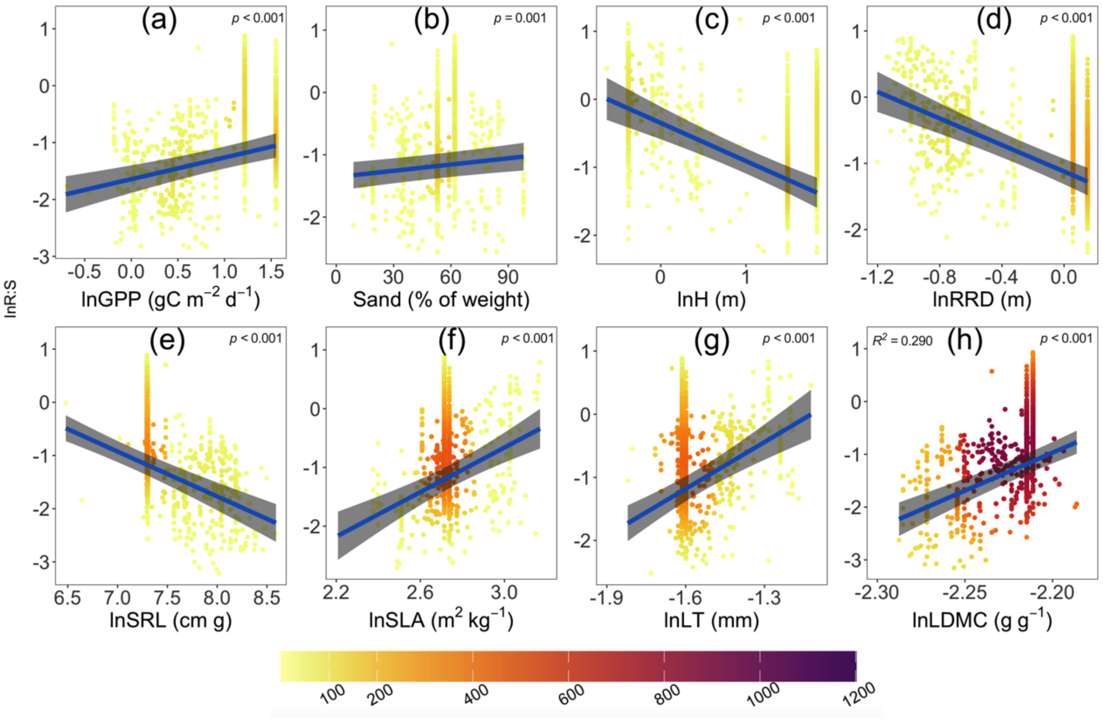

Fig. S3 Partial residual plots from the regression of natural log-transformed root-shoot biomass ratio (R:S) against explanatory variables in shrub species. The plots are from the ordinary least-squares multiple linear regression in Table S1. a: log-transformed gross primary production (ln GPP, gC m^{-2} d^{-1}). b: soil sand content (Sand, % of weight). c: log-transformed vegetation height (H, m). d: log-transformed root rooting depth (ln RRD, m). e: log-transformed specific root length (ln SRL, cm g). f: log-transformed specific leaf area (ln SLA, m^2 kg^{-1}). g: leaf thickness (LT, mm). h: log-transformed leaf dry matter content (ln LDMC, g g^{-1}). Extreme outliers (0.25% lowest and 0.25% highest) were removed to improve visualization, while all statistical analyses retain the complete dataset. Coefficients and standard errors for the fitted lines are given in Table S1.

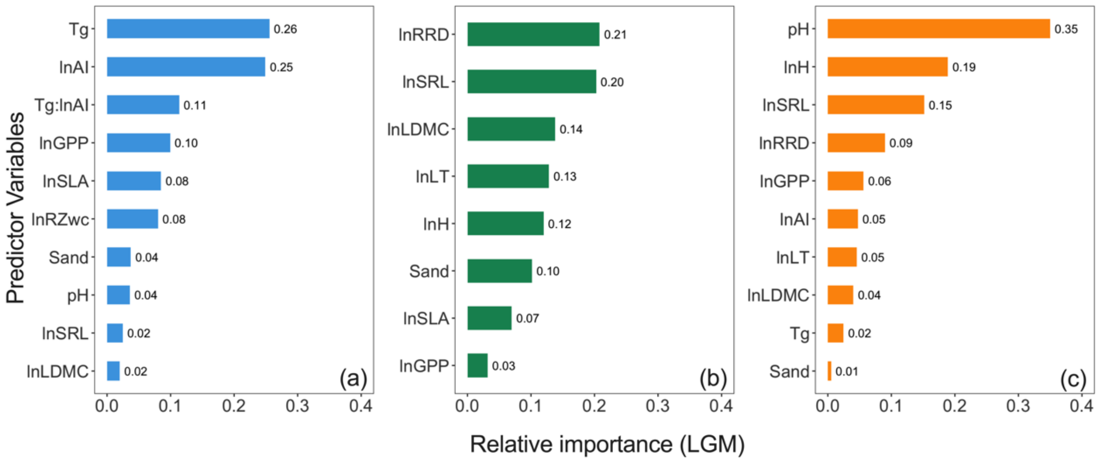

Fig. S4 Importance of climate and traits explanatory variables from regression models in forests, shrubs and herbaceous species. a-c, Predictive relative importance for forests (a), shrubs (b) and herbaceous (c). Numbers represent relative importance as indicated by the Lindeman–Merenda–Gold (LMG) statistic.

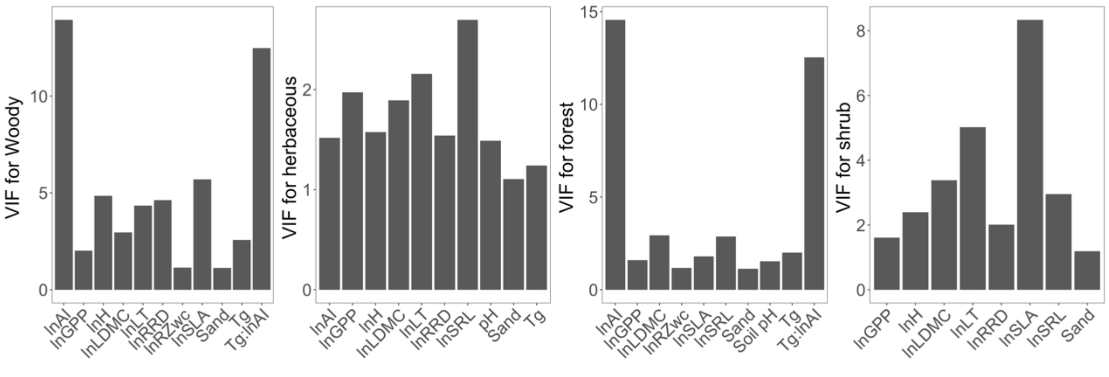

Fig. S5 Variance Inflation Factors (VIF) for different predictors. VIFs for the model with predictors in different plant types. The variables comprise growing-season mean temperature (T_g, ˚C), log-transformed root zone water capacity (ln RZ_{WC}, mm), log-transformed gross primary production (ln GPP, gC m^{-2} d^{-1}), soil pH (unitless), soil sand content (Sand, % of weight), log-transformed aridity index (ln AI, unitless) and the interaction between T_g and ln AI (T_g * lnAI). natural log-transformed vegetation height (ln H, m), natural log-transformed leaf thickness (ln LT, mm), natural log-transformed leaf dry matter content (ln LDMC, g g^{-1}), natural log-transformed specific leaf area (ln SLA, m^2 kg^{-1}), natural log-transformed specific root length (ln SRL, cm g^{-1}) and natural log-transformed root rooting depth (ln RRD, m).

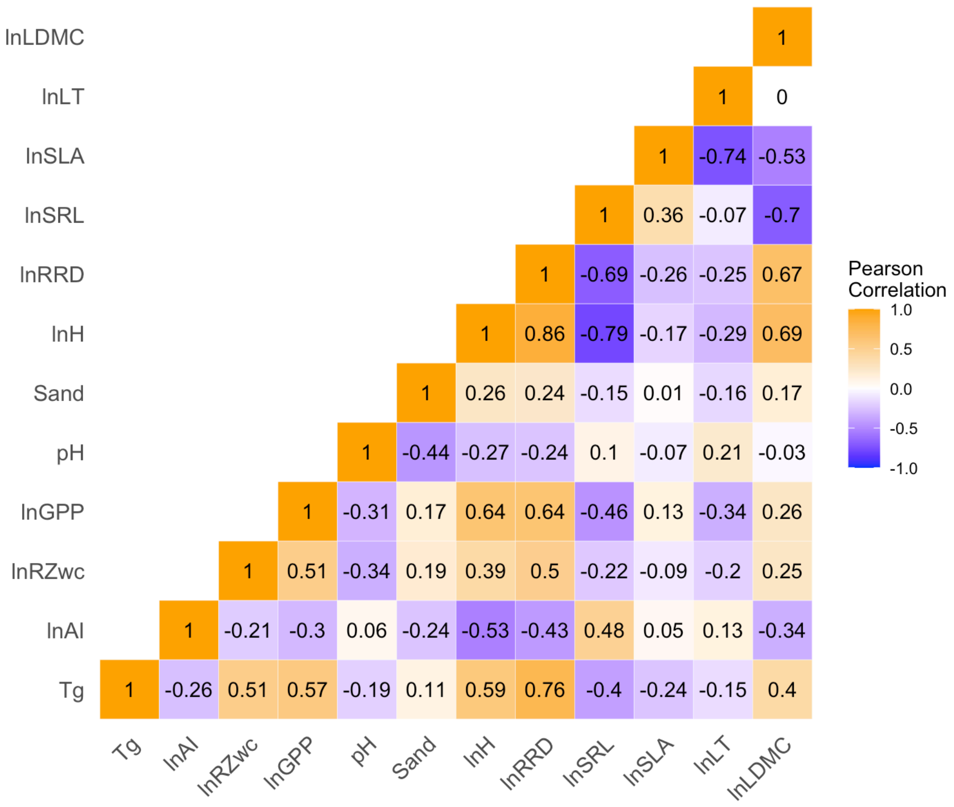

Fig. S6 Pearson correlation coefficients between predictors. The coefficients are shown by values and gradient of colours for growing-season mean temperature (T_g, ˚C), log-transformed root zone water capacity (ln RZ_{WC}, mm), log-transformed gross primary production (ln GPP, gC m^{-2} d^{-1}), soil pH (unitless), soil sand content (Sand, % of weight) and log-transformed aridity index (ln AI, unitless). Vegetation height (H, m), leaf thickness (LT, mm), leaf dry matter content (LDMC, g g^{-1}), specific leaf area (SLA, m^2 kg^{-1}), specific root length (SRL, cm g^{-1}) and root rooting depth (RRD, m).

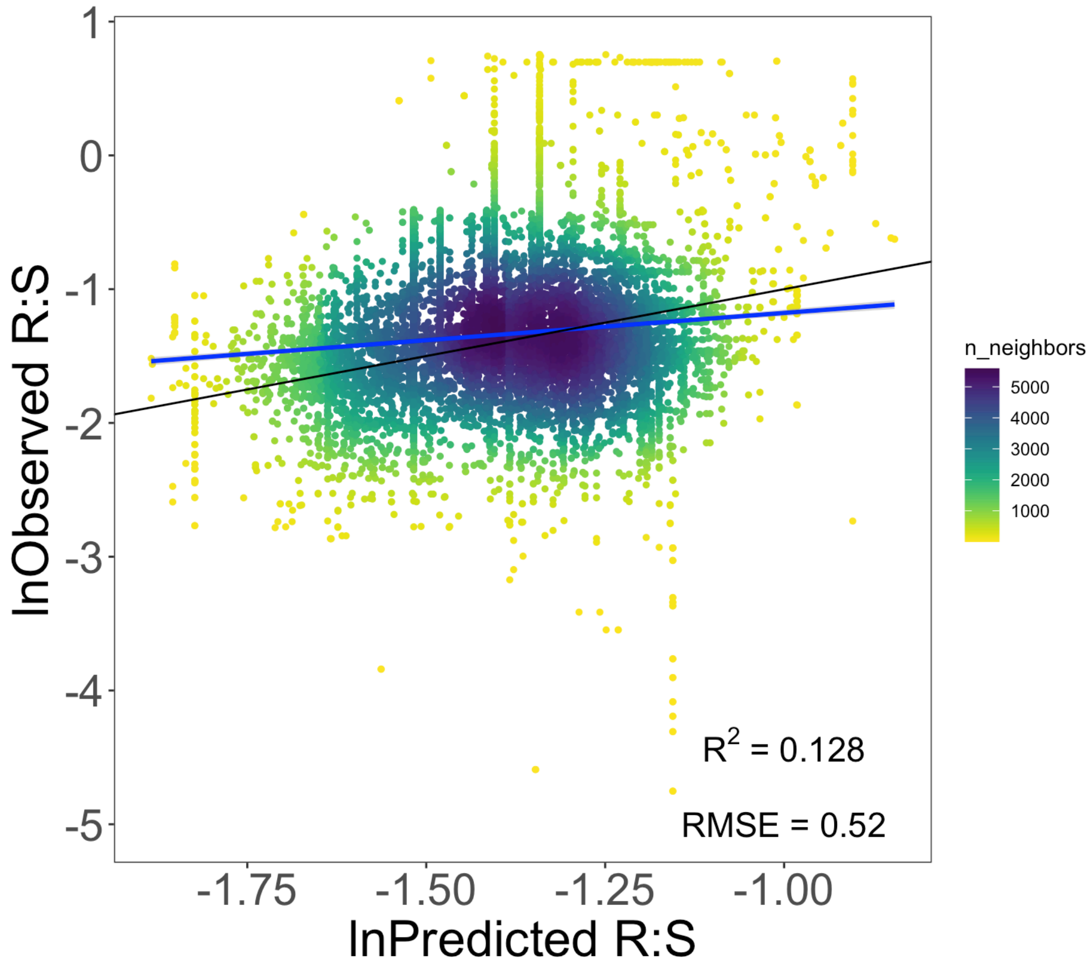

Fig.S7 Observed and predicted trends of R:S based on the multiple regression model in woody species. Predicted relationships for woody vegetation are shown in blue with 95% confidence intervals in grey. The black line represents the 1:1 line.

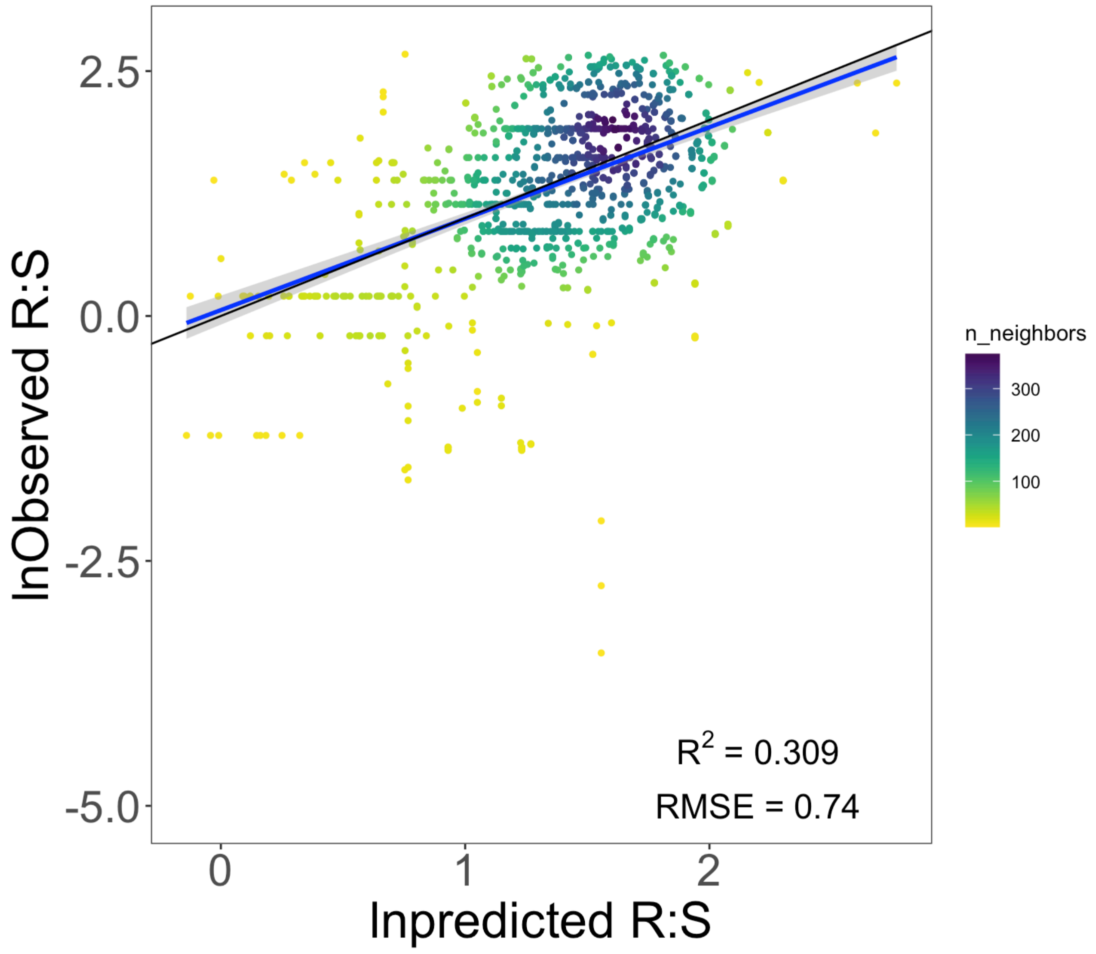

Fig.S8 Observed and predicted trends of R:S based on the multiple regression model in herbaceous species. Predicted relationships for woody vegetation are shown in blue with 95% confidence intervals in grey. The black line represents the 1:1 line.

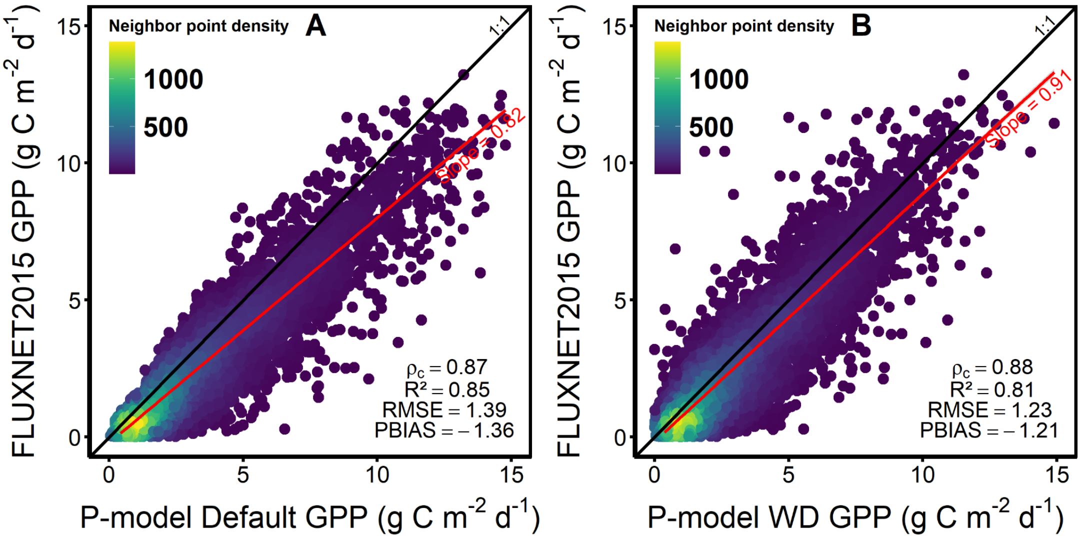

Fig.S9 8-day mean GPP (n = 6,005) comparison between 72 sites in the FLUXNET2015 dataset and P-model estimates: a) using the P-model calibrated soil-water stress (Stocker *et al.*, 2020), and; b) the water deficit method approach presented in this study (Eq S1). The red line is the linear regression. For the neighbour density we used the *ggpointdensity* R package (Kremer, 2019), which aims to estimate how many points are overlapping.

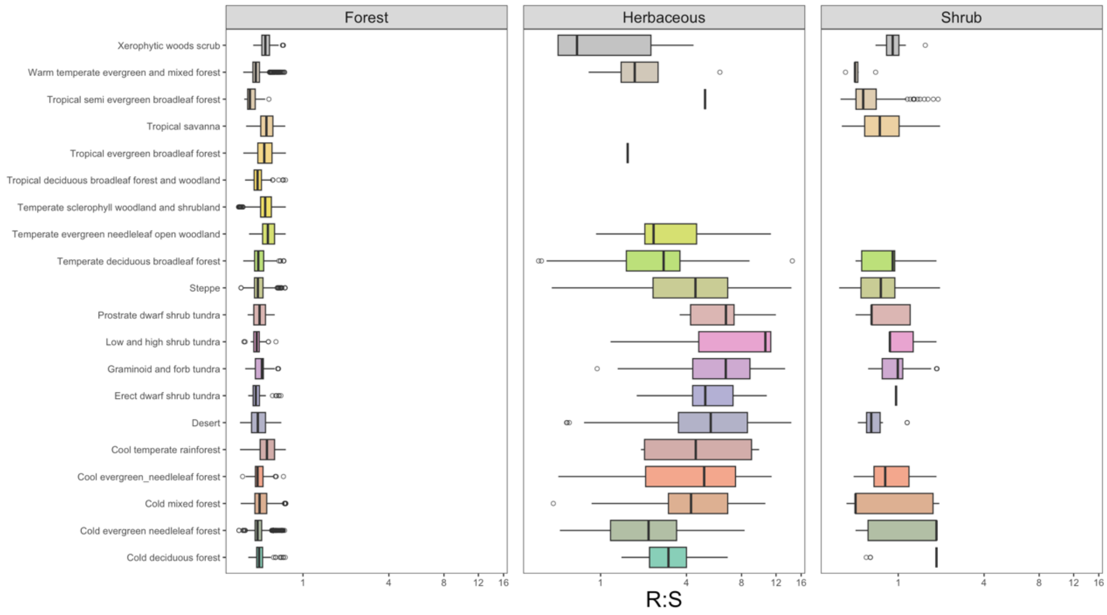

Fig. S10. Boxplot of R:S ratios across Potential Natural Vegetation (PNV) types, grouped by vegetation structural categories (Forest, Shrub, and Herb). Each box represents the distribution of R:S observations within a specific PNV formation (e.g., tropical evergreen broadleaf forest, steppe, tundra). Boxes show the interquartile range (IQR), with the median indicated by a horizontal line with 1.5× IQR, and points beyond represent outliers.

References

Cucchi, M., Weedon, G. P., Amici, A., Bellouin, N., Lange, S., Schmied, H. M., … & Buontempo, C. (2020). WFDE5: bias adjusted ERA5 reanalysis data for impact studies. *Earth System Science Data, 12*(3), 2097–2120. https://doi.org/10.5194/essd-12-2097-2020

Hijmans, R. J. (2020). Geographic data analysis and modeling. R package version 3.4–5. https://CRAN.R-project.org/package=raster

Kremer, L. P. M. (2019). A cross between a 2D density plot and a scatter plot. R package version 0.1.0. https://CRAN.R-project.org/package=ggpointdensity

Nóbrega, R. L. B., Sandoval, D., Miranda, R., Tan, S., & Prentice, I. C. (2025). Ecosystem root-zone water capacity is driven by water deficit and precipitation seasonality. Preprint (Version 1). *Research Square*. https://www.researchsquare.com/

Pastorello, G., Trotta, C., Canfora, E., Chu, H., Christianson, D., Cheah, Y. W., … & Law, B. (2020). The FLUXNET2015 dataset and the ONEFlux processing pipeline for eddy covariance data. *Scientific Data, 7*(1), 225. https://doi.org/10.1038/s41597-020-0534-3

Stocker, B. D., Wang, H., Smith, N. G., Harrison, S. P., Keenan, T. F., Sandoval, D., … & Prentice, I. C. (2020). P-model v1.0: An optimality-based light use efficiency model for simulating ecosystem gross primary production. *Geoscientific Model Development, 13*(3), 1545–1581. https://doi.org/10.5194/gmd-13-1545-2020

Walsh, R. P. D., & Lawler, D. M. (1981). Rainfall seasonality: Description, spatial patterns and change through time. *Weather, 36*(7), 201–208. https://doi.org/10.1002/j.1477-8696.1981.tb05400.x
